# TIPred: a novel stacked ensemble approach for the accelerated discovery of tyrosinase inhibitory peptides

**DOI:** 10.1186/s12859-023-05463-1

**Published:** 2023-09-21

**Authors:** Phasit Charoenkwan, Sasikarn Kongsompong, Nalini Schaduangrat, Pramote Chumnanpuen, Watshara Shoombuatong

**Affiliations:** 1 Modern Management and Information Technology, College of Arts, Media and Technology, Chiang Mai University, Chiang Mai, 50200 Thailand; 2 Interdisciplinary Graduate Program in Bioscience, Faculty of Science, Kasetsart University, Bangkok, 10900 Thailand; 3 Center for Research Innovation and Biomedical Informatics, Faculty of Medical Technology, Mahidol University, Bangkok, 10700 Thailand; 4 Department of Zoology, Faculty of Science, Kasetsart University, Bangkok, 10900 Thailand; 5 Omics Center for Agriculture, Bioresources, Food, and Health, Kasetsart University (OmiKU), Bangkok, 10900 Thailand

**Keywords:** Tyrosinase inhibitory peptides, Sequence analysis, Bioinformatics, Machine learning, Feature selection, Stacking strategy

## Abstract

**Background:**

Tyrosinase is an enzyme involved in melanin production in the skin. Several hyperpigmentation disorders involve the overproduction of melanin and instability of tyrosinase activity resulting in darker, discolored patches on the skin. Therefore, discovering tyrosinase inhibitory peptides (TIPs) is of great significance for basic research and clinical treatments. However, the identification of TIPs using experimental methods is generally cost-ineffective and time-consuming.

**Results:**

Herein, a stacked ensemble learning approach, called TIPred, is proposed for the accurate and quick identification of TIPs by using sequence information. TIPred explored a comprehensive set of various baseline models derived from well-known machine learning (ML) algorithms and heterogeneous feature encoding schemes from multiple perspectives, such as chemical structure properties, physicochemical properties, and composition information. Subsequently, 130 baseline models were trained and optimized to create new probabilistic features. Finally, the feature selection approach was utilized to determine the optimal feature vector for developing TIPred. Both tenfold cross-validation and independent test methods were employed to assess the predictive capability of TIPred by using the stacking strategy. Experimental results showed that TIPred significantly outperformed the state-of-the-art method in terms of the independent test, with an accuracy of 0.923, MCC of 0.757 and an AUC of 0.977.

**Conclusions:**

The proposed TIPred approach could be a valuable tool for rapidly discovering novel TIPs and effectively identifying potential TIP candidates for follow-up experimental validation. Moreover, an online webserver of TIPred is publicly available at http://pmlabstack.pythonanywhere.com/TIPred.

**Supplementary Information:**

The online version contains supplementary material available at 10.1186/s12859-023-05463-1.

## Background

Tyrosinase is a metalloenzyme that possesses a copper binding domain which is conserved across different organisms including fruits, vegetables, fungi, mammals, and insects that utilize it for cuticle sclerosis and wound healing [2, 3]. The enzyme catalyzes the transformation of tyrosine, an amino acid, into DOPA (dihydroxyphenylalanine), which is subsequently converted into melanin—the pigment responsible for determining skin, hair, and eye color [1, 4]. Moreover, this enzyme also plays a role in the biosynthesis of other pigments such as dopamine and norepinephrine [2]. Overproduction of melanin and instability of tyrosinase activity could cause several hyperpigmentation disorders, which are the conditions that result in excessive skin pigmentation and cause darker, discolored patches on the skin [3]. These disorderes can be caused by a variety of factors, including sun exposure, hormonal changes, inflammation, genetics, and certain medications [4, 5]. Examples of common hyperpigmentation disorders include melasma, age spots, and post-inflammatory hyperpigmentation [4]. Treatment options for hyperpigmentation disorders include topical lightening agents, chemical peels, and laser therapy. In some cases, reducing exposure to triggers and protecting the skin from further sun damage can also help reduce the appearance of hyperpigmentation [4–6].

Substances that can hinder the function of the enzyme tyrosinase are known as tyrosinase inhibitors, and are frequently utilized in skin lightening products aimed at reducing the visibility of hyperpigmentation and dark spots on the skin [5]. Some natural tyrosinase inhibitors include kojic acid, arbutin, and licorice extract [7, 8]. On the other hand, chemical tyrosinase inhibitors include hydroquinone, azelaic acid, and glycolic acid [9, 10]. However, these chemical whitening agents may lead to various undesirable side effects such as skin irritation, allergic reactions, sensitivity to sunlight, discoloration, and exogenous ochronosis [11–14]. Tyrosinase inhibitory peptides (TIPs) refer to short chains of amino acids, usually comprised of 3–20 units, that are capable of impeding the activity of the tyrosinase enzyme. This enzyme is responsible for the synthesis of melanin, the pigment that determines skin color [17]. Recently, bioactive peptides have become an increasingly popular medicinal agent, and TIPs derived from food sources are highly favored due to their excellent biological safety and ease of absorption. These peptides are viewed as a promising alternative to chemical tyrosinase inhibitors like hydroquinone [17–19]. In addition to TIPs, amino acids released during digestion in the gastrointestinal tract can also be completely absorbed even without ingestion [15]. The clinical trials for novel TIPs derived from various animal and plant sources are currently underway [16–18].

Therefore, the identification of TIPs through the use of sequence information is crucial to accelerate their implementation in clinical settings. In this regard, machine learning (ML) techniques have been explored to facilitate the high-throughput discovery of new TIPs. Currently, only one computational method has been developed for the identification of TIPs. This method was introduced by Kongsompong et al. [19]. Specifically, this group employed two popular ML methods (random forest (RF) and k-nearest neighbour (KNN)) trained with three interpretable feature descriptors (amino acid composition (AAC), physicochemical properties (PCP), and dipeptide composition (DPC)). These KNN and RF classifiers were trained and evaluated on the dataset consisting of 133 TIPs and 13 non-TIPs. The performance of KNN and RF classifiers were 0.97 and 0.99, respectively, in terms of accuracy (ACC) on the independent test dataset. Although Kongsompong’s method provides a high prediction performance, their method has a few flaws and needs to be improved. Firstly, the method was developed using a small number of negative samples. Hence, their performance in non-TIP identification might not be satisfactory. Secondly, this study did not offer a comparative analysis of the impact of well-known feature encodings and ML algorithms on TIP prediction. Thirdly, this study did not provide a web server.

**Fig. 1:**
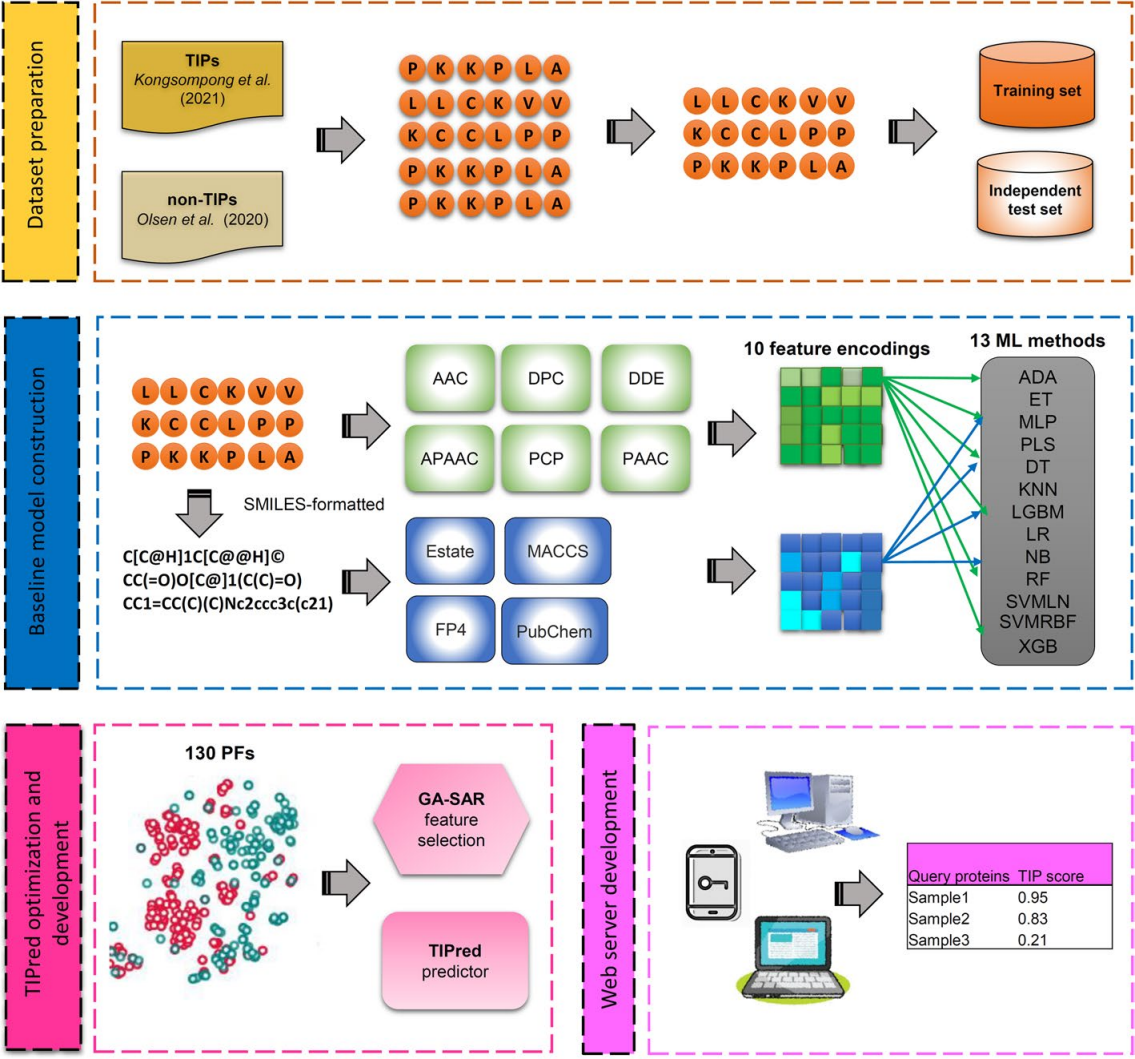
System flowchart of the proposed TIPred. The overall workflow for the development of TIPred contains four major steps: dataset preparation, baseline model construction, TIPred optimization, and web server development

Considering these limitations, we introduce TIPred for the large-scale identification of TIPs by using only peptide sequence information. The design and development of TIPred is summarized in Fig. 1. Major contributions of this study are listed as follows:

(i) To the best of our knowledge, TIPred is the first stacked ensemble approach developed for the identification and characterization of TIPs.
(ii) TIPred employed different feature encoding schemes from multiple aspects, including, amino acid composition, chemical structure properties, physicochemical properties and pseudo-amino acid composition, integrated state-of-the-art ML classifiers to develop a more stable meta-model. In addition, we investigated the contributions of different types of feature encodings in TIP prediction.
(iii) The independent test results indicated that TIPred achieved a better performance compared to the existing method and several conventional ML classifiers in terms of ACC (0.923), Matthew’s correlation coefficient (MCC) (0.744) and area under the receiver operating characteristics (ROC) curve (AUC) (0.964).
(iv) In TIPred, we utilized an interpretable Shapley Additive exPlanation (SHAP) approach to provide a better understanding of the functional mechanisms of TIPs.
(v) TIPred-assisted virtual screening approach was introduced and used for the accelerated discovery of novel TIPs.

## Materials and methods

### Construction of training and independent datasets

In this study, the positive dataset containing 133 TIPs was derived from the study of Kongsompong et al. [19]. These TIPs are peptides that have been experimentally verified as having tyrosinase inhibitory properties. Until now, there has been no source of experimentally verified non-TIPs. Therefore, to establish a dependable dataset, the non-antioxidative peptides obtained from Olsen et al. [20] were utilized to create the negative dataset in this study. TIPs usually exhibit dual activities, they can inhibit tyrosinase through the photoaging system by binding to the catalytic domain, as well as directly scavenge free radicals as antioxidants [15, 18, 21–23]. Thus, we selected peptides that were empirically confirmed as non-effective in both categories of antioxidant properties (i.e., free radical scavengers and iron chelators) as negative samples. After removal of duplicated sequences, 287 non-TIPs were obtained. Finally, the benchmark dataset contained 133 TIPs and 287 non-TIPs. Among these, 106 TIPs and 230 non-TIPs were randomly selected to construct the training dataset (called TIP-TRN), the remaining peptides were used to construct the independent test dataset (called TIP-IND).

### Feature encoding methods

To encode the TIPs and non-TIPs, we utilized 10 different feature encoding schemes, which are categorized into multiple groups, namely amino acid composition (AAC, DPC, and DDE), pseudo-amino acid composition (APAAC and PAAC), physicochemical properties (PCP) and chemical structure properties (Estate, FP4, MACCS, and PubChem). Among these feature encoding schemes, AAC, APAAC, DDE, DPC, PAAC, and PCP, which are known as sequence-based feature descriptors, can be used to encode FASTA-formatted TIPs and non-TIPs into fixed-length feature vectors by considering the 20 standard amino acids along with the *iFeature* Python package [24–26]. By using the remaining feature encoding schemes, the FASTA-formatted TIPs and non-TIPs were converted into their corresponding chemical structures (SMILES format) by using the *RDKit* software [27]. Then, the Chemistry Development Kit (CDK) was used to encode SMILES-formatted TIPs and non-TIPs into fixed-length feature vectors [26, 28–31]. Details of all the 10 feature encodings are summarized in Table 1.

**Table 1:** Summary of ten different feature encodings along with their corresponding description and dimension

Order	Descriptors	Description	Dimension	References
1	AAC	Frequency of 20 amino acids	20	[59, 60]
2	APAAC	Amphiphilic pseudo-amino acid composition	22	[61]
3	DDE	Dipeptide deviation from expected mean	400	[24]
4	DPC	Frequency of 400 dipeptides	400	[59, 60]
5	PCP	Different biochemical and biophysical properties extracted from the AAindex database	11	[59, 60]
6	PAAC	Pseudo amino acid composition	21	[61]
7	Estate	Electrotopological state atom types	79	[29, 30, 62]
8	FP4	Presence of SMARTS patterns for functional groups	307	[29, 30, 63]
9	MACCS	Binary representation of chemical features defined by MACCS keys	166	[29, 30, 64]
10	Pubchem	Binary representation of substructures defined by PubChem	881	[29, 30, 65]

### Feature selection technique

Training a prediction model with high-dimensional input feature vectors can cause overfitting and underfitting issues. In this context, the feature selection method is needed to enhance the prediction performance and optimize computational time [28–30, 32]. In this study, we used our proposed genetic algorithm (GA-SAR) for constructing an optimal feature set containing *m* useful features [25, 26, 33]. In 2019, Charoenkwan et al. initially introduced this method for the interpretable identification of quorum sensing peptides [33]. Until now, the GA-SAR method has been applied for the prediction and characterization of many protein and peptide functions [25, 26, 34, 35]. In brief, the chromosomes of the GA-SAR consist of two main genes, namely binary gene and parametric gene. The chromosomes and gene of the GA-SAR are referred as GA-chrom and GA-gene herein, respectively. Herein, the parameters and their values for the GA-SAR consist of *m*_*start*_ = 5, *m*_*end*_ = 20, *P*_*m*_ = 0.05, and *Pop* = 50. Detailed report regarding this algorithm is provided in our previous studies [25, 26, 33] along with the Additional file 1 [25, 26, 33].

### The architecture of the proposed model TIPred

Herein, TIPred was developed by using the stacking strategy. Stacking is well-known as a powerful ensemble learning approach that is able to automatically combine multiview information derived from different ML classifiers as means to create a more accurate and stable predictor [25, 26, 29, 30]. Thus, the development of our proposed TIPred involves two main steps, including (1) baseline model construction and (2) meta-model development.

In the first part, we encoded the TIP-TRN dataset using 10 types of feature encodings, including DPC, PAAC, PCP, AAC, DDE, APAAC, FP4, Estate, PubChem, and MACCS, in combination with 13 ML methods, including ADA, ET, MLP, PLS, DT, KNN, LGBM, LR, NB, RF, SVMLN, SVMRBF and XGB, for the baseline model development. Specifically, the baseline models were created based on a wide range of feature encodings from multiple perspectives, including amino acid composition, chemical structure properties, pseudo-amino acid composition, and physicochemical properties [36–40]. In total, 130 baseline models were trained and constructed by using the scikit-learn package (Table 2). In addition, we conducted a comprehensive assessment of all the 130 baseline models in TIP prediction by performing both cross-validation and independent tests. Herein, the best-performing baseline model was indicated by using the Matthew's Correlation Coefficient (MCC) on the TIP-TRN dataset.

**Table 2:** Parameter search details used for the construction of nine ML-based classifiers

Method^a^	Parameters	Range of parameters
ADA	n_estimators	[20, 50, 100, 200, 500]
ET	n_estimators	[20, 50, 100, 200, 500]
LGBM	n_estimators	[20, 50, 100, 200, 500]
LR	Cost	[0.001, 0.01, 0.1, 1, 10, 100]
MLP	hidden_layer_sizes	[20, 50, 100, 200, 500]
RF	n_estimators	[20, 50, 100, 200, 500]
SVMLN	Cost	[2^0^ to 2^5^] in log_2_ steps
SVMRBF	Cost	[2^−4^ to 2^4^] in log_2_ steps
XGB	n_estimators	[20, 50, 100, 200, 500]

^a^ADA: AdaBoost, DT: decision tree, ET: extremely randomized trees, KNN: k-nearest neighbor, LGBM: light gradient boosting machine, LR: logistic regression, MLP: multilayer perceptron, NB: naive Bayes, PLS: partial least squares, RF: random forest, SVMRBF: support vector machine with radial basis function, SVMLN: support vector machine with linear kernels, XGB: extreme gradient boosting

In the second part, we generated a new probabilistic feature vector (PFV) of 130 dimension (130-D) by using 130 probabilistic features (PFs) derived from all the 130 baseline models and tenfold cross-validation scheme, where PFs were the predicted confidence of TIPs. Then, the 130-D feature vector was used to develop the meta-model based on PLS (called mPLS) [25]. Although the 130-D feature vector contains only 130 PFs, some of these PFs involve redundant and noisy information. Thus, the GA-SAR was used to establish an optimal feature set containing *m* useful PFs. Specifically, the GA-SAR’s chromosome used herein involved *n* = 130 features. As a result, the GA-chrom contains 130 binary GA-genes (${f}_{i}$). If the *i*^*th*^ PP is considered as a useful feature when ${f}_{i}=1$; otherwise, the *i*th feature is not considered. Finally, the feature set exhibiting the highest cross-validation MCC was deemed as the optimal one. Furthermore, additional evaluation metrics, including AUC, ACC, MCC, balanced accuracy (BACC), sensitivity (Sn), and specificity (Sp), were selected to evaluate the effectiveness of our proposed model. The descriptions of these evaluation metrics can be found in the Additional file 1 [40, 41].

### Screening novel TIPs

In this study, our proposed model was employed to perform a large-scale identification of TIPs from the putative hempseed (*Cannabis sativa*) trypsinized peptidome derived from a previous study [42]. Only 73 unique peptides with the proper amino acid length (10–57) were consider for our analysis. The putative peptides from *Cannabis sativa* seed having the highest probabilistic scores were deemed as candidate TIPs. After that, the molecular docking approach was used to assess the ability of the selected TIPs to bind to the active site of the tyrosinase enzyme. Specifically, the molecular docking between the selected TIPs and the polyphenol oxidase domain (chains A–D) of the crystal structure of mushroom tyrosinase from *Agaricus bisporus* (PDB: 2Y9X) was performed using two protein-peptide docking web servers, namely [43] GalaxyPepDock (http://galaxy.seoklab.org/pepdock) and HPEPDOCK (http://huanglab.phys.hust.edu.cn/hpepdock/). The GalaxyPepDock server was used to conduct the template-based molecular docking simulation, while the HPEPDOCK server was used to estimate the template-free (global) molecular docking scores.

## Results and discussion

### Investigation of the contribution of different machine learning methods and feature encodings

**Fig. 2:**
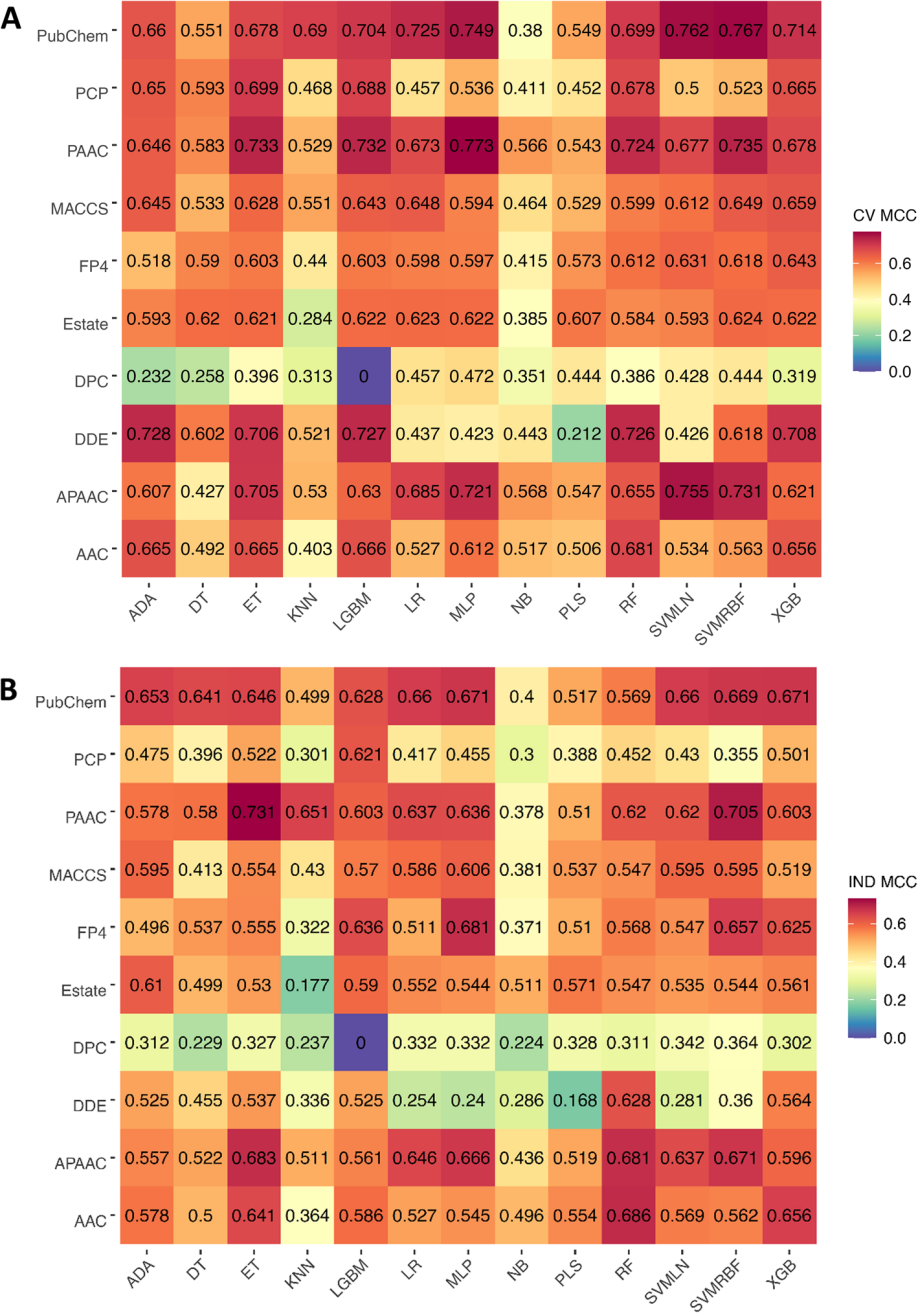
MCC values of 130 baseline models in terms of tenfold cross-validation (**A**) and independent (**B**) tests

In this section, we investigated the contribution of different types of feature encodings in TIP prediction. Thus, all the 10 feature encodings were assessed pairwise using all the 13 ML methods in terms of tenfold cross-validation and independent tests. Figure 2 and Additional file 1: Tables S1-S3 detail the predictive performance of the 130 different ML classifiers. Additional file 1: Table S3 shows that the highest average MCC of 0.664 is achieved by using PubChem, while the second and third highest average MCC of 0.661 and 0.629 were achieved by using PAAC and APAAC, respectively. And, we noticed that there were ten PubChem-based (range 0.660–0.767), nine PAAC-based (range 0.646–0.773), nine APAAC-based (range: 0.607–0.755) classifiers with MCC greater than 0.6 (see Fig. 2). Interestingly, all the top ten ML classifiers were developed based on PubChem, PAAC, and APAAC, including MLP-PAAC, SVMRBF-PubChem, SVMLN-PubChem, SVMLN-APAAC, MLP-PubChem, SVMRBF-PAAC, ET-PAAC, LGBM-PAAC, and SVMRBF-APAAC. This demonstrates that these feature encodings could be beneficial for TIP prediction. Although it could be noticed that MLP-PAAC attained the highest performance in terms of ACC (0.882) and MCC (0.767) on the TIP-TRN dataset, this classifier failed to achieve a better performance on the TIP-IND dataset, with ACC of 0.870, MCC of 0.636, and AUC of 0.960. This evidence indicates that the performance of a single feature-based models is not stable on the TIP-IND dataset. To address this issue, we were motivated to generate a more comprehensive and reliable model by using the stacking strategy.

### Performance evaluation of TIPred

Herein, we utilized the stacked strategy to create a stacked model by using PLS method in conjunction with the 130-D feature vector generated from multiple baseline models. To maximize the utility of the 130-D feature vector, this feature vector was optimized by using the GA-SAR as means to determine the optimal number (*m*) of PFs. In this study, the optimal number of PFs was 11 (or the 11-D feature vector). Specifically, the 11-D feature vector was generated by the baseline models of ET-DDE, MLP-PubChem, XGB-PubChem, SVMRBF-APAAC, NB-DDE, ADA-FP4, NB-Estate, LR-MACCS, SVMRBF-FP4, MLP-FP4, and PLS-PubChem. The performance of the 130-D and 11-D feature vectors are recorded in Table 3. As seen in Table 3, MCC, ACC, Sn, and Sp of the 11-D feature vector are 0.920, 0.958, 0.945, and 0.973, which are 13.21, 6.58, 6.55, and 6.64%, respectively, higher than the 130-D feature vector in terms of the tenfold cross-validation test. Furthermore, in case of the independent test results, the 11-D feature vector still achieved the overall best performance compared to the 130-D feature vector. In this context, we utilized the 11-D feature vector to build our proposed model, TIPred.

**Table 3:** Cross-validation and independent test results for the control and optimal model

Evaluation strategy	Feature	Number of feature	ACC	BACC	Sn	Sp	MCC	AUC
Cross-validation	AFV	130	0.869	0.869	0.870	0.868	0.741	0.942
	BFV	11	0.916	0.917	0.917	0.917	0.837	0.955
Independent test	AFV	130	0.909	0.948	1.000	0.895	0.725	0.989
	BFV	11	0.923	0.956	1.000	0.912	0.757	0.977

### The Stacking model is capable of improving the predictive performance

**Fig. 3:**
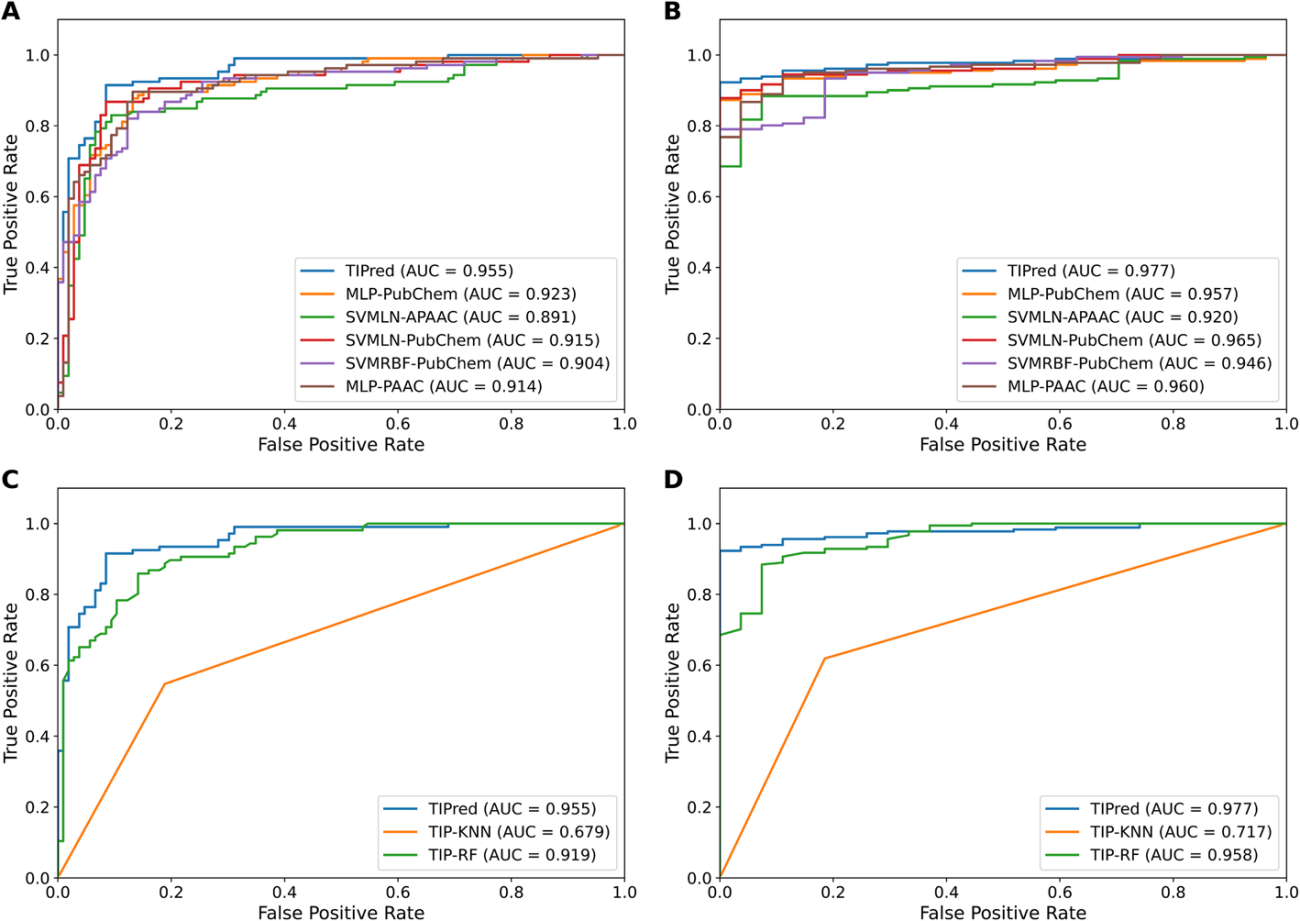
Performance comparison of TIPred with related methods in terms of tenfold cross-validation (**A**, **C**) and independent (**B**, **D**) tests. (**A**, **B**) ROC curves of TIPred and top five baseline models. (**C**, **D**) ROC curves of TIPred and existing methods

In this section, we aim to highlight the improved performance provided by the stacking strategy, by comparing the performance of TIPred with BLAST-based predictors and the top five baseline models (i.e., MLP-PubChem, SVMLN-APAAC, SVMLN-PubChem, SVMRBF-PubChem, and MLP-PAAC). Additional file 1: Table S4 presents the independent test results of BLAST-based predictors with various *E*-values. It is worth noting that the highest MCC of 0.406 was achieved using an *E*-value cutoff value of 0.1. However, the Sn of this optimal cutoff value was unsatisfactory (Sn of 0.185), while Sn of other cutoff values were in the range of 0.037–0.111. This demonstrated that the BLAST-based predictor was not capable of precisely identifying true TIPs. As can be seen from Fig. 3 and Table 4, TIPred outperformed the top five baseline models in terms of all performance metric on the TIP-IND dataset. Specifically, the BACC, Sn, Sp, and AUC of TIPred were 0.959, 1.000, 0.912, and 0.757, which were 4.61, 3.70, 5.52, and 12.06%, respectively, higher than that of the best-performing baseline model (MLP-PAAC). This indicates that the stacked ensemble learning approach is indeed effective in improving the performance of TIP prediction.

**Table 4:** Performance comparison of TIPred and top five ML classifiers

Evaluation strategy	Method	ACC	BACC	Sn	Sp	MCC	AUC
Cross-validation	MLP-PubChem	0.873	0.872	0.850	0.895	0.749	0.923
	SVMLN-APAAC	0.873	0.874	0.916	0.831	0.755	0.891
	SVMLN-PubChem	0.878	0.877	0.887	0.866	0.762	0.915
	SVMRBF-PubChem	0.882	0.881	0.869	0.894	0.767	0.904
	MLP-PAAC	0.882	0.884	0.871	0.897	0.773	0.914
	TIPred	0.916	0.917	0.917	0.917	0.837	0.955
Independent test	MLP-PubChem	0.889	0.921	0.963	0.878	0.671	0.957
	SVMLN-APAAC	0.880	0.899	0.926	0.873	0.637	0.920
	SVMLN-PubChem	0.875	0.928	1.000	0.856	0.660	0.965
	SVMRBF-PubChem	0.880	0.931	1.000	0.862	0.669	0.946
	MLP-PAAC	0.870	0.910	0.963	0.856	0.636	0.960
	TIPred	0.923	0.956	1.000	0.912	0.757	0.977

### TIPred outperforms the existing method

To demonstrate the effectiveness of the model, it is necessary to compare the proposed model TIPred with the existing method [19]. As the existing method did not provide a webserver, we implemented KNN-based and RF-based classifiers by strictly utilizing the same procedure reported in the study of Kongsompong et al. [19] using the same training dataset. Table 5 illustrates that our proposed model, TIPred, achieved the best performance as judged by five out of six evaluation metrics (i.e., ACC, BACC, Sn, Sp, MCC, and AUC) on both the TIP-TRN and TIP-IND datasets. Specifically, the BACC, Sn, and MCC values achieved by TIPred were 8.98, 18.52, and 11.33% higher than RF-based classifier in terms of the TIP-IND dataset. Altogether, based on both the cross-validation and independent test results, TIPred consistently demonstrated a stable performance, indicating its effectiveness and robustness. In addition, the higher values of Sp and MCC in terms of the TIP-IND dataset are sufficient to elucidate that TIPred could effectively reduce the number of false positives, which plays a crucial role for minimizing the experimental costs and burden.

**Table 5:** Performance comparison of TIPred and the existing predictors

Evaluation strategy	Method	ACC	BACC	Sn	Sp	MCC	AUC
Cross-validation	TIP-KNN	0.680	0.679	0.811	0.547	0.383	0.679
	TIP-RF	0.845	0.845	0.821	0.869	0.695	0.919
	TIPred	0.916	0.917	0.917	0.917	0.837	0.955
Independent test	TIP-KNN	0.644	0.717	0.815	0.619	0.294	0.717
	TIP-RF	0.904	0.866	0.815	0.917	0.643	0.958
	TIPred	0.923	0.956	1.000	0.912	0.757	0.977

### Feature importance analysis

**Fig. 4:**
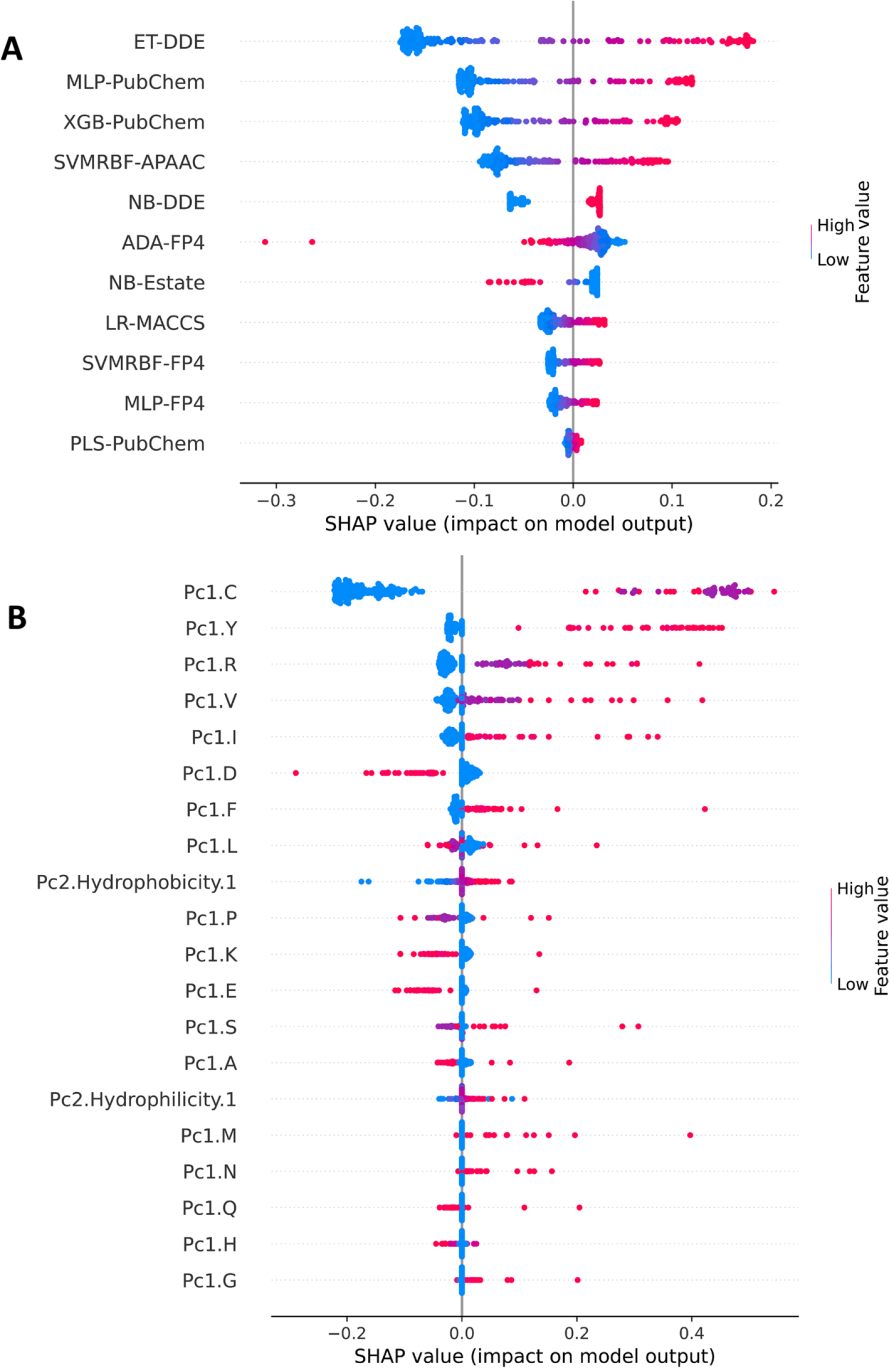
Feature importance from TIPred **(A)** and SVMRBF-APAAC **(B)** as ranked by SHAP values based on the training dataset. Color indicates the magnitude and direction of the contribution of features to TIPred and SVMRBF-APAAC for TIP prediction, where positive and negative SHAP values indicate the probability that the prediction outputs are positive and negative samples, respectively

The SHAP framework is well-known as an interpretable and powerful framework used to provide information about how features can affect the output of the model. Therefore, we utilized this framework to analyze the prediction outputs of the proposed TIPred and its baseline models. Figure 4A and Additional file 1: Fig. S1A demonstrate the impact of the 11 PFs on the prediction of TIPred, where the positive and negative SHAP values indicate the probability that the prediction outputs are relatively positive and negative classes, respectively. We obtained the top-eight informative PFs with the highest SHAP values from eight baseline models of SVMLN-PubChem, ET-DDE, SVMRBF-APAAC, MLP-PubChem, LGBM-PubChem, XGB-MACCS, PLS-Estate, and PLS-APAAC (refer to Fig. 4A and Additional file 1: Table S5). Taking SVMLN-PubChem as an example, peptide sequences with high PF values of SVMLN-PubChem have a high probability of being TIPs. On the other hand, peptide sequences with high PF values of PLS-Estate have a high possibility of being non-TIPs. Among the top-eight informative PFs, SVMRBF-APAAC was found to be the fourth-best informative PF. Figure 4B along with Additional file 1: Fig. S1B and Table S6 display the impact of top 20 informative features on the prediction of SVMRBF-APAAC. Based on the SHAP values, we noticed that the ten top-ranked features consist of Cys, Tyr, Arg, Val, Ile, Asp, Phe, Leu, hydrophobicity, and Pro. As shown in Fig. 4B, Cys, Tyr, Arg, Val, Ile, Phe, and hydrophobicity are abundant in TIPs compared to non-TIPs, while Asp, Leu, and Pro are abundant in non-TIPs compared to TIPs.

The amino acid composition findings are consistent with previous reports, indicating that TIPs tend to contain higher levels of certain amino acids, including Cys, Tyr, Arg, Phe, and Met [44–49]. Some of the well-known TIPs were rich in Ser, Trp, Arg, and sulfur-containing amino acids (Cys and Met). These characteristics are typical of well-known peptides that inhibit tyrosinase and chelate metals. The sulfur-containing amino acids, Cys and Met, have been found to be associated with tyrosinase inhibition and copper chelation in natural TIPs derived from hydrolyzed rice-bran-derived albumin [47]. Schurink et al. [42] observed that peptides with polar, uncharged amino acids, particularly Cys, are effective tyrosinase inhibitors that have a high reductive effect on the melanin synthesis pathway. According to research, Cys plays a role in limiting tyrosinase activity by converting o-quinone intermediates into stable colorless cysteine-quinone adducts, lowering polyphenol precursor levels and preventing the formation of polymeric melanin products. Cys-containing peptides can also compete with catalytically active copper ions, preventing them from binding to tyrosinase [48, 49].

Interestingly, hydrophobicity, a feature of physicochemical property has also been found in the ten top-ranked features together with other amino acids. There is also supporting evidence suggesting that hydrophobic amino acid residues can enhance tyrosinase inhibition. The hydrophobic nature of TIPs, including amino acids such as Phe, Trp, Met, and particularly Ala, has been found to play an important role in inhibiting melanogenesis [45]. Furthermore, it was observed that the aromatic amino acid Phe has the ability to stabilize free radicals through electron donation and maintain its antioxidant stability through its resonant structure [46–48]. The peptides containing the cationic amino acid Arg have been found to possess remarkable activity in chelating copper ions and generally exhibit excellent tyrosinase binding properties, owing to the presence of the guanidine group [42]. Interestingly, the dipeptide Arg-Lys, which has been reported as the active composition in the TIPs fraction of hydrolyzed rice albumin [47], was not among the top ten SHAP values identified in this study.

### TIPred-assisted virtual screening for novel TIPs identification

**Fig. 5:**
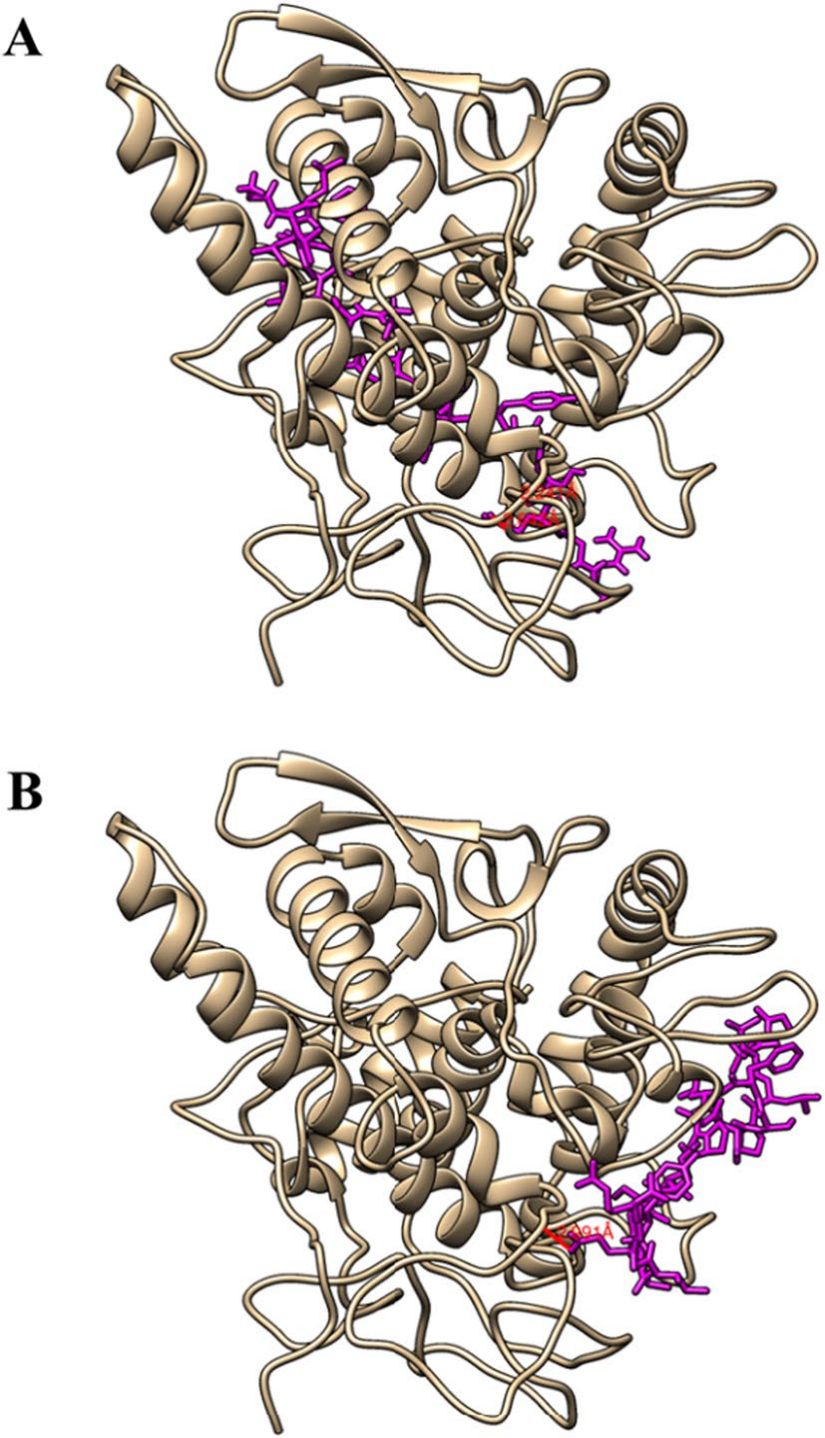
Molecular docking of E2–12 to the tyrosinase (PDB: 2Y9X) generated from GalaxyPepDock (**A**) and HPEPDOCK (**B**). The tyrosinase is shaded in gold, while the peptide sequences and hydrogen bonds are shown in pink and red, respectively

Herein, we applied TIPred-assisted virtual screening approach for the identification of novel TIPs. To showcase TIPs prediction and screening, hempseed (*Cannabis sativa*) peptidome was the most suitable choice due to the fact that it is a high-protein plant source (20–25% content) that has been extensively utilized in the development of numerous products for the cosmetics, therapeutic, functional food, and nutraceutical industries [50, 51]. Hempseed protein hydrolysates have been recognized as a valuable source of bioactive peptides with various health-promoting effects [52]. As a result, hempseed peptides have garnered attention for their potential bioactive pharmaceutical properties, including antioxidant and tyrosinase inhibitory abilities. Additional file 1: Table S7 lists the probabilistic scores of the 73 putative peptides from *Cannabis sativa* seed. As mentioned above, we selected the candidate TIPs in terms of the probabilistic score and considered as potential TIPs. The top five TIPs with a probabilistic score of 1.000 were identified, including A-2 (ISSSTLALFAALMLVAHAVAFR), E1–9 (YTIQQNGLHLPSYTNTPQLVYIVK), E2–12 (GLLLPSFLNAPMMFYVIQGR), E3–38 (NAMYAPQYTMNAHNIIYAIR), and E3–6 (LTIQPNGLHLPSYTNGPQLIHVIR). This suggests that these peptides are highly likely to have TIP activity and can be considered as potential TIPs for further validation. To further demonstrate the effectiveness of these top-five potential TIPs, we performed molecular docking between these TIPs and the polyphenol oxidase domain (chain A–D) of the crystal structure of mushroom tyrosinase by using by GalaxyPepDock and HPEPDOCK (Fig. 5 and Additional file 1: Figures S2-S6). The calculated binding affinity between the TIP candidates and tyrosinase was represented by molar Gibbs free energy (ΔG) and the equilibrium dissociation constant (Kd). As shown in Table 6, the scores (ΔG, Kd, and molecular docking score) of the top-five potential TIPs were − 11.6 to − 9.4 kcal/mol, 3.1E−09 to 1.6E−07, and − 201.2040 to 134.0631 kJ/mol, respectively. Among the top-five potential TIPs, E2–12 outperformed other peptides in terms of molecular docking score. To be specific, the ΔG, Kd, and molecular docking score of E2–12 were − 10.1, 4.0E−08, and − 201.2040, respectively.

**Table 6:** Calculated binding affinity (∆G), dissociation constant (Kd), and binding energy scores from the molecular docking results from HPEPDOCK of csTIPs and P4 to the tyrosinase (2Y9X chainD) based on the PROGIDY and PIMA web servers

Name	ΔG (kcal/mol)	Kd at 25.0 ℃	H-bond ener. (kJ/mol)	Elec. ener. (kJ/mol)	VDW. ener. (kJ/mol)	Molecular docking score (kJ/mol)
A-2	− 9.4	1.4E−07	− 5.4749	− 5.7960	145.3340	134.0631
E1–9	− 11.6	3.1E−09	− 3.1403	1.8997	− 90.3478	− 91.5884
E2–12	− 10.1	4.0E−08	− 12.6447	− 6.7877	− 181.7720	− 201.2040
E3–6	− 11.0	8.3E−09	− 4.5694	4.2906	− 141.8230	− 142.1020
E3–38	− 10.8	1.2E−08	− 13.4909	0.0000	− 48.8050	− 62.2959
P4	− 9.3	1.6E−07	− 34.2300	24.0025	− 9.2737	− 19.5013

**Fig. 6:**
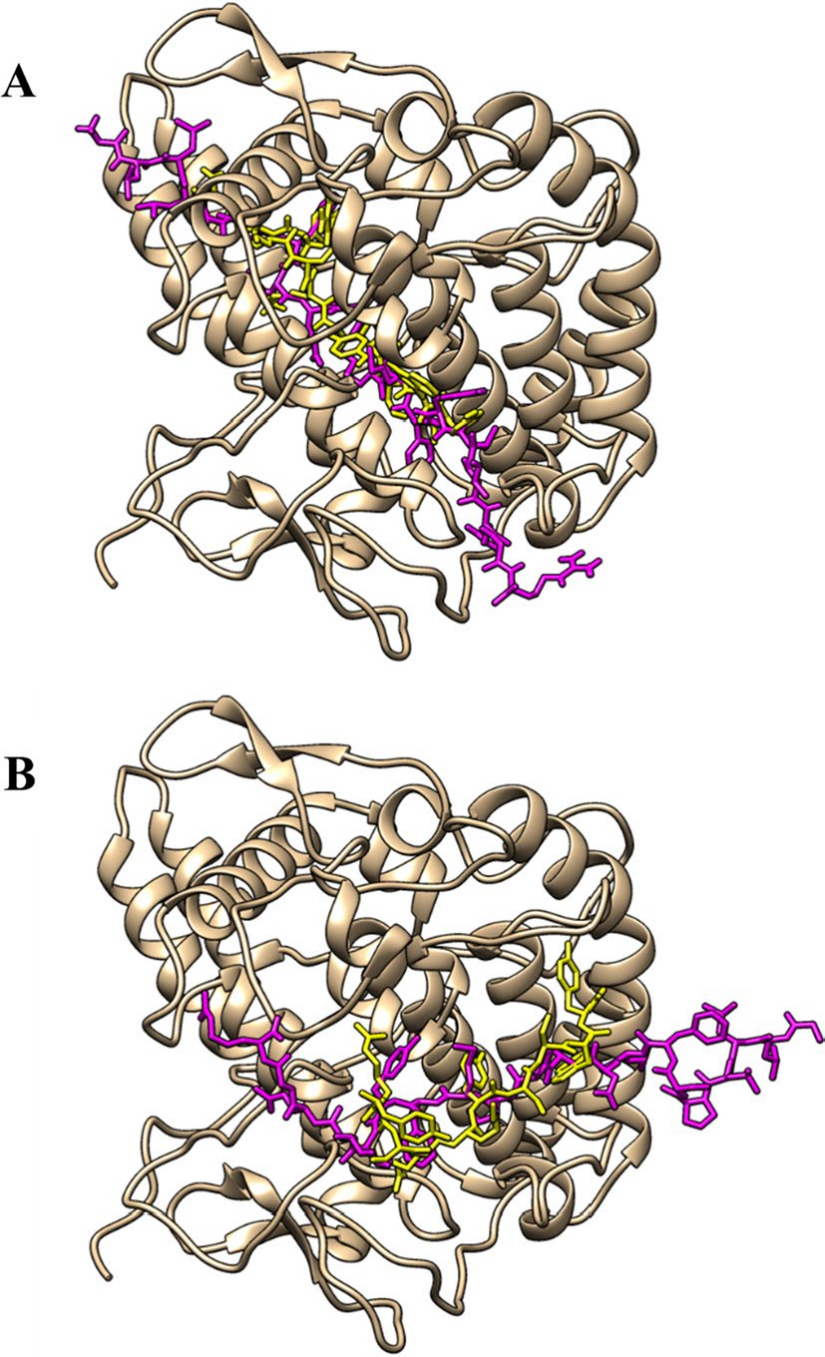
Comparative molecular docking of the highest molecular docking scored csTIP candidate (E2–12) and the positive control peptide (P4) on the crystal structure of tyrosinase (PDB: 2Y9X) from different protein-peptide docking tools: GalaxyPepDock (**A**) and HPEPDOCK (**B**). The structure of the tyrosinase is shaded in gold, while E2–12 and P4 are shown in pink and yellow, respectively

To indicate the effectiveness of E2–12, we compared its performance with the commercial TIP, i.e., P4 (YRSRKSSWP) or decapeptide-12. There have been multiple studies that indicate P4 as the most well-known peptide that is currently being used as the primary active component in the LumixylTM skin brightening product [17, 53]. The common binding position of the highest molecular docking scored csTIP candidate (E2–12) and the positive control peptide (P4) was revealed by the comparative molecular docking on the crystal structure of tyrosinase (Fig. 6). The molecular docking result could confirm the overlapped interacting regions on the active site of tyrosinase structure on both protein-peptide docking methods (GalaxyPepDock and HPEPDOCK). Table 6 indicates that E2–12 exhibited a better potential inhibition of tyrosinase as compared to the commercial TIP P4 in terms of ΔG (− 10.1 versus − 9.3), Kd (4.0E−08 versus 1.6E−07), and molecular docking score (− 201.2040 versus − 19.5013). Furthermore, all the hydrogen bonds between peptides and tyrosinase proposed by the molecular docking simulation are listed in Additional file 1: Table S8. According to the molecular docking simulation experiments, the distance in the hydrogen bond between E2–12 and tyrosinase structures was 1.5–3.3 Å, implying that E2–12 could be deemed as a moderately strong covalent interaction [54]. Similar to the analyzed results of abalone biomimetic peptides (hdTIPs) [43], the cationic amino acid residues (Arg20, 38, 95, 268, 321) of the catalytic domain on tyrosinase seems to be the key binding target of E2–12 and other hempseed TIPs. These results indicate that the peptide E2–12 as derived from this study could be a promising TIP. Altogether, the virtual screening result of TIPred is adequate to demonstrate that TIPred has the potential to be a useful and efficient tool for quickly screening and identifying promising TIPs.

## Conclusion

This study introduces a novel stacked ensemble approach (termed TIPred) for the accurate and high-throughput identification of TIPs. TIPred combines a comprehensive set of feature encoding schemes from multiple aspects, such as chemical structure properties, physicochemical properties, and composition information, with 13 well-known ML methods to create a more stable model. The experimental results of both the tenfold cross-validation and independent tests indicate the effectiveness of our stacked model TIPred, outperforming the existing method and several conventional ML classifiers. The improved performance of TIPred can be attributed to several factors: (1) The integration of different feature encodings can provide more discriminative patterns; (2) The GA-SAR methods can determine the optimal number of features as a means of reducing the overfitting issue and improving the performance; and (3) The stacking strategy can effectively maximize the utilization of baseline models to obtain a more accurate TIP identification. Our new method is anticipated to contribute to community-wide efforts in screening and identifying potential TIP candidates for the treatment of skin pigmentation disorders and other clinical applications. Although TIPred has achieved better and more robust performance, it still has some limitations, which can be addressed in future work. One possible extension is to collect additional TIPs to develop a more comprehensive prediction model. Another extension could be the employment of well-known feature extractors, such as a bidirectional recurrent neural network (RNN) [55] and ProtBERT [56], to effectively capture the key information of TIPs. For the last extension, we can try to incorporate TIPred with recent innovative computational frameworks, such as an iterative feature representation algorithm [57] and deep learning (DL)-based framework [39, 58].

### Supplementary Information

**Additional file 1.** Supplementary Figures and Tables.

## Data Availability

All the data used in this study are available at http://pmlabstack.pythonanywhere.com/TIPred.

## Abbreviations

DOPA: Dihydroxyphenylalanine
TIPs: Tyrosinase inhibitory peptides
RF: Random forest
KNN: K-nearest neighbour
AAC: Amino acid composition
PCPs: Physicochemical properties
DPC: Dipeptide composition
MCC: Matthew’s correlation coefficient
ROC: Receiver operating characteristics
AUC: Area under the receiver operating characteristics curve
SHAP: Shapley Additive exPlanation
CDF: Chemistry development kit
ML: Machine learning
ET: Extremely randomized trees
SVM: Support vector machine
ACC: Accuracy
DT: Decision tree
LGBM: Light gradient boosting machine
LR: Logistic regression
MLP: Multilayer perceptron
NB: Naive Bayes
PLS: Partial least squares
SVMRBF: Support vector machine with radial basis function
SVMLN: Support vector machine with linear kernels
XGB: Extreme gradient boosting.
PF: Probabilistic feature
GA: Genetic algorithm
SAR: Self-assessment-report operation
Sp: Specificity
Sn: Sensitivity
TP: True positive
FP: False positive
TN: True negative
FN: False negative
APAAC: Amphiphilic pseudo-amino acid composition
DDE: Dipeptide deviation from expected mean
PAAC: Pseudo amino acid composition
ADA: AdaBoost
RNN: Recurrent neural network
DL: Deep learning
