# Supplementary material for: TIPred: a novel stacked ensemble approach for the accelerated discovery of tyrosinase inhibitory peptides
Source: BMC Bioinformatics. 2023 Sep 21;24:356. doi: 10.1186/s12859-023-05463-1 (PMC10512532; doi:10.1186/s12859-023-05463-1)
Supplement: Supplementary file 1 — Additional file 1. Supplementary Figures and Tables. [file 12859_2023_5463_MOESM1_ESM.docx]

# **TIPred: a novel stacked ensemble approach for the accelerated discovery of tyrosinase inhibitory peptides**

Phasit Charoenkwan^1^, Sasikarn Kongsompong^2^, Nalini Schaduangrat^3^, Pramote Chumnanpuen^4,5,*^, Watshara Shoombuatong^3,*^

^1^ Modern Management and Information Technology, College of Arts, Media and Technology, Chiang Mai University, Chiang Mai, Thailand, 50200.

^2^ Interdisciplinary Graduate Program in Bioscience, Faculty of Science, Kasetsart University, Bangkok, Thailand, 10900

^3^ Center of Data Mining and Biomedical Informatics, Faculty of Medical Technology, Mahidol University, Bangkok, Thailand, 10700.

^4^ Department of Zoology, Faculty of Science, Kasetsart University, Bangkok, Thailand, 10900.

^5^ Omics Center for Agriculture, Bioresources, Food, and Health, Kasetsart University (OmiKU), Bangkok,Thailand, 10900.

*Corresponding author: [pramote.c@ku.th](mailto:pramote.c@ku.th) (**PM**), [watshara.sho@mahidol.ac.th](mailto:watshara.sho@mahidol.ac.th). (**WS**)

### **Feature selection based on GA-SAR**

There exists six major steps for selecting *m* import features based on the GA-SAR method:

(i) Randomly construct a population of *Pop* individuals;

(ii) Assess the performance of all *Pop* individuals by using the fitness function and 10-fold cross-validation scheme;

(iii) Employ a tournament selection to determine the best-performing one to create a mating pool;

(iv) Determine two parents from the mating pool to conduct using the SAR mutation operator;

(v) Use the SAR mutation operator to randomly select *Pop* individuals from new population; (vi) Stop if the stopping condition is satisfied; otherwise go to the 2^nd^ step.

Detailed information regarding this algorithm is provided in our previous studies [1-3].

### **Performance evaluation**

Here, the performance of TIPred and other ML classifiers were assessed by using five standard performance measures, including AUC, Mathew’s correlation coefficient (MCC), accuracy (ACC), specificity (Sp) and sensitivity (Sn) [4-6]. MCC, ACC, Sp and Sn are generally defined as follows:

| $MCC=\frac{TP\times TN-FP\times FN}{\sqrt{(TP+FP)(TP+FN)(TN+FP)(TN+FN)}}$ | (1) |
| --- | --- |
| $ACC=\frac{TP+TN}{\left( TP+TN+FP+FN \right)}$ | (2) |
| $Sp=\frac{\mathrm{TN}}{\left( TN+FP \right)}$ | (3) |
| $Sn=\frac{\mathrm{TP}}{\left( TP+FN \right)}$ | (4) |
| $BACC=(Sn+Sp)\times0.5$ | (5) |

where the number of correctly predicted true TIPs and true non-TIPs are referred to as TP and TN, respectively. On the other hand, FP stands for the number of non-TIPs that are predicted to be TIPs, and FN stands for the number of TIPs that were predicted to be non-TIPs [7-12].

### **Supplementary Figures**


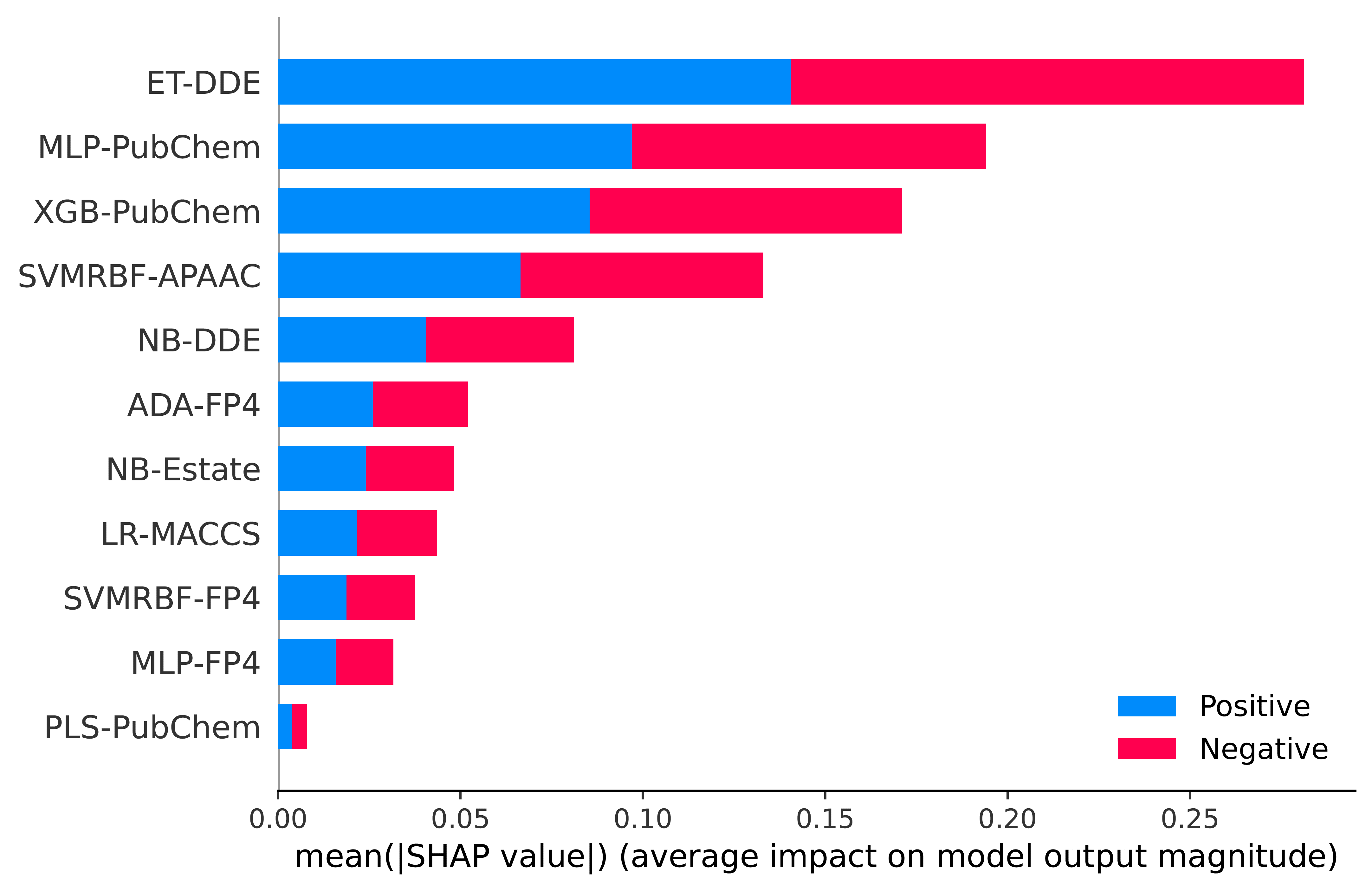


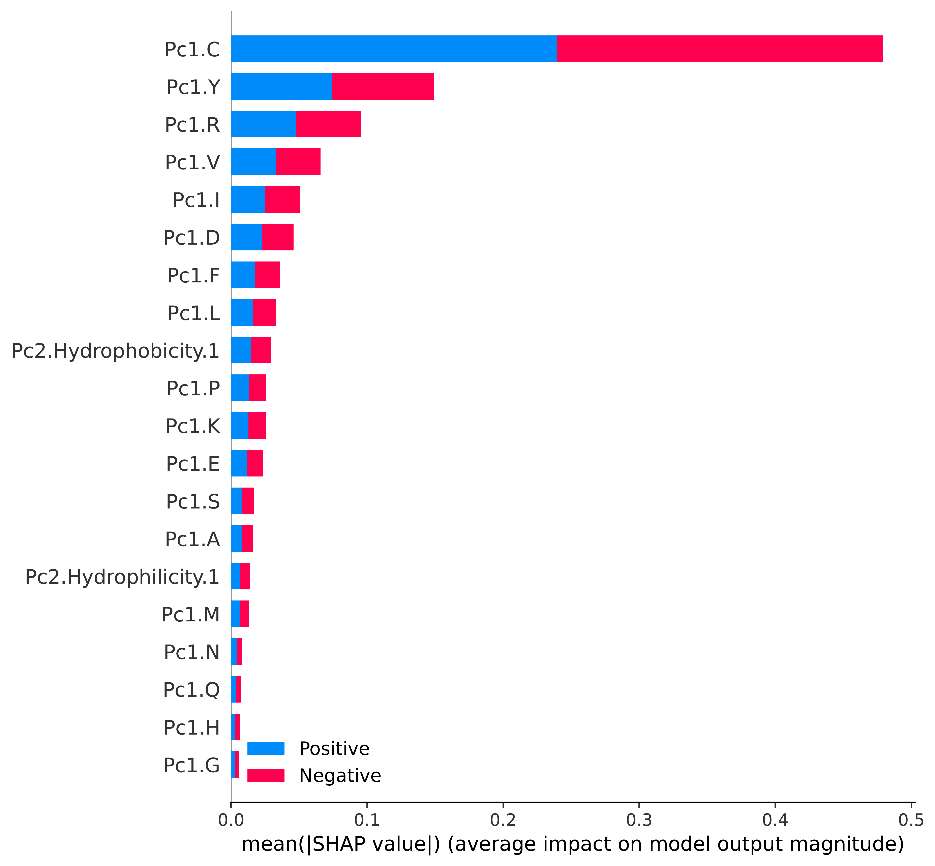


**Figure S1** SHAP summary plot of features from TIPred **(A)** and SVMRBF-APAAC **(B)** as ranked by SHAP values based on the training dataset.

**
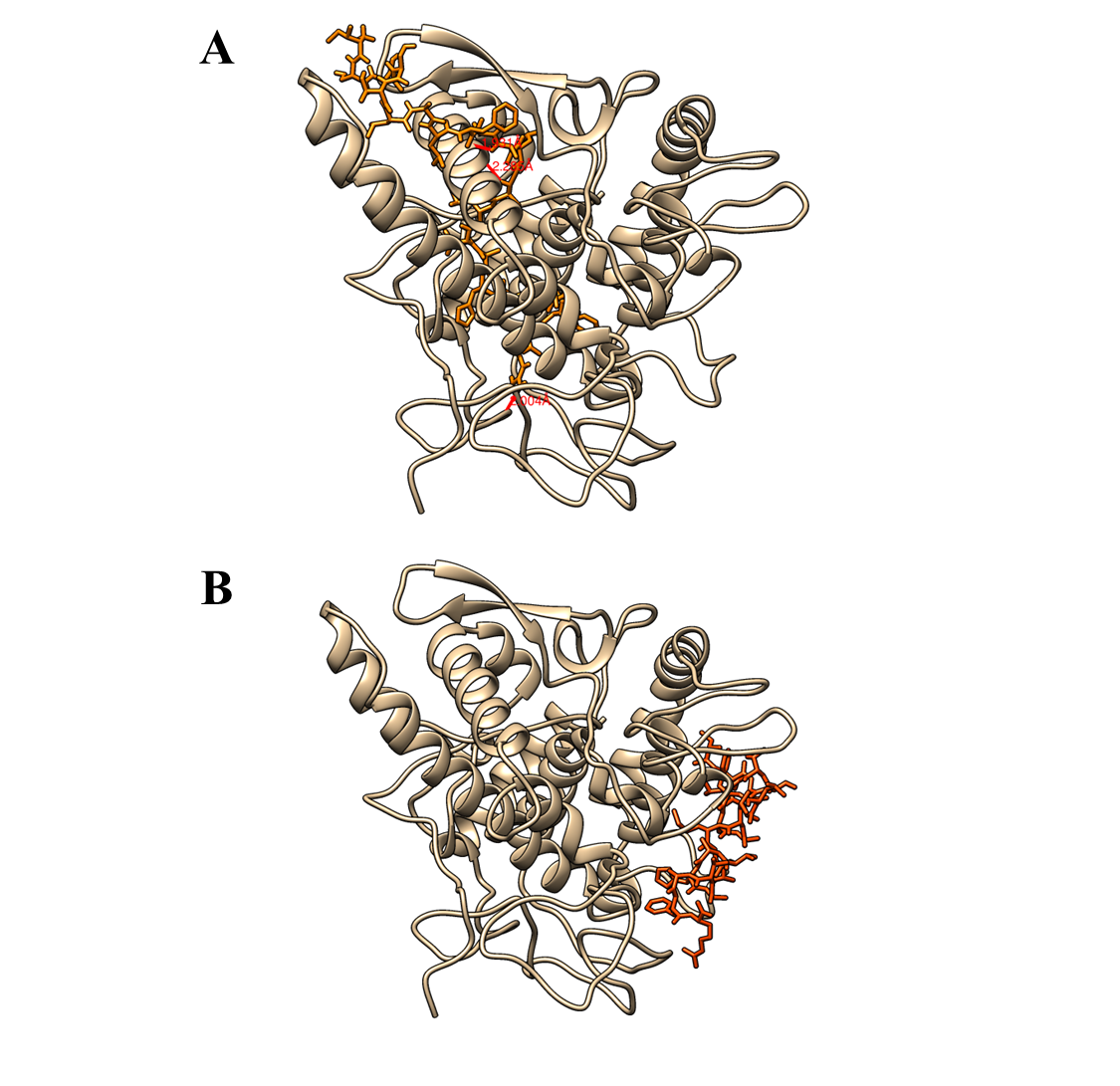
**

**Figure S2** Molecular docking of A-2 peptide to the tyrosinase (PDB: 2Y9X) from GalaxyPepDock (A) and HPEPDOCK (B). The tyrosinase is shaded in gold, and the peptide sequences are colored in orange. The hydrogen bonds are shown as red lines.


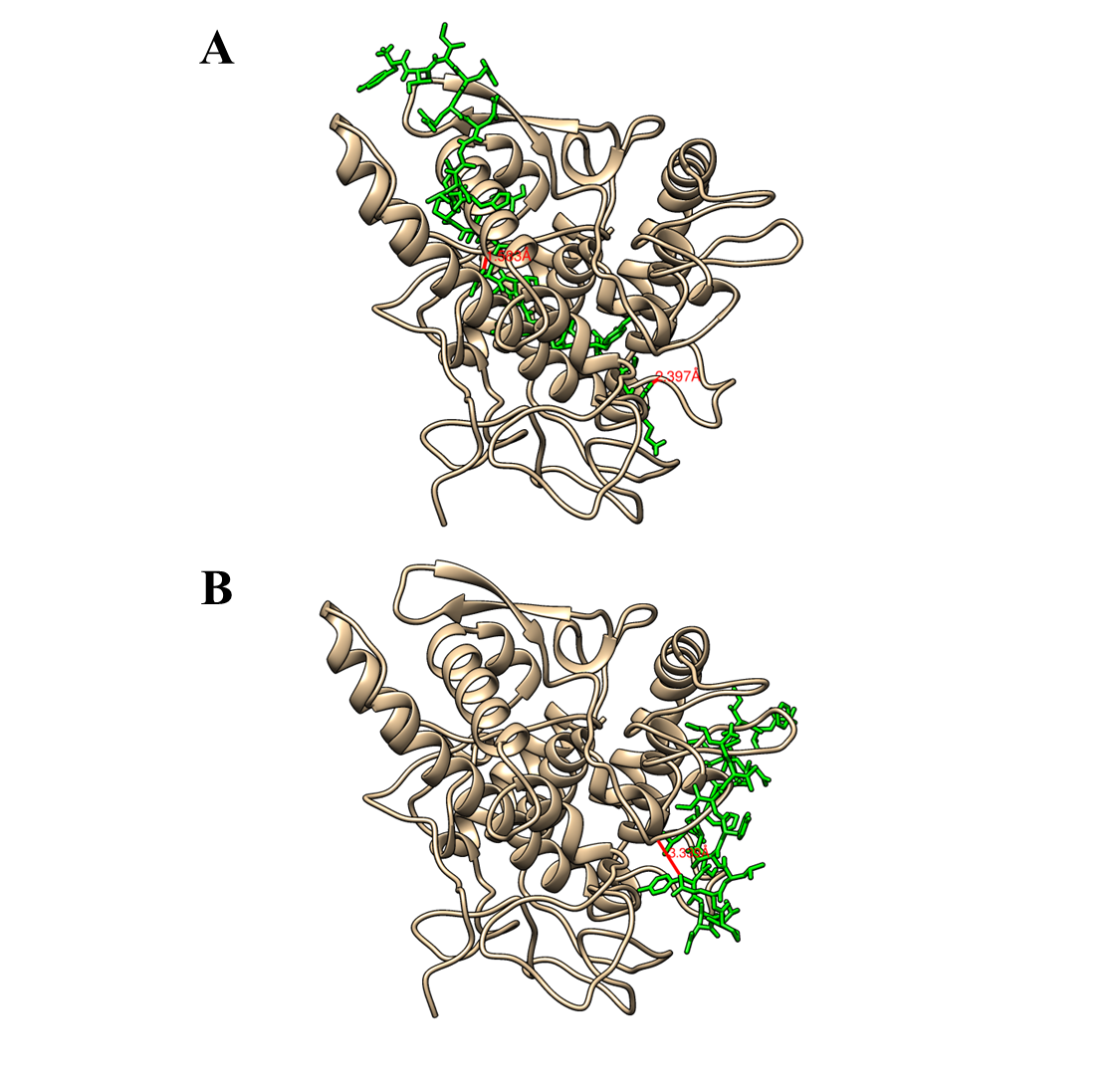


**Figure S3** Molecular docking of E1-9 peptide to the tyrosinase (PDB: 2Y9X) from GalaxyPepDock (A) and HPEPDOCK (B). The tyrosinase is shaded in gold, and the peptide sequences are colored in green. The hydrogen bonds are shown as red lines.


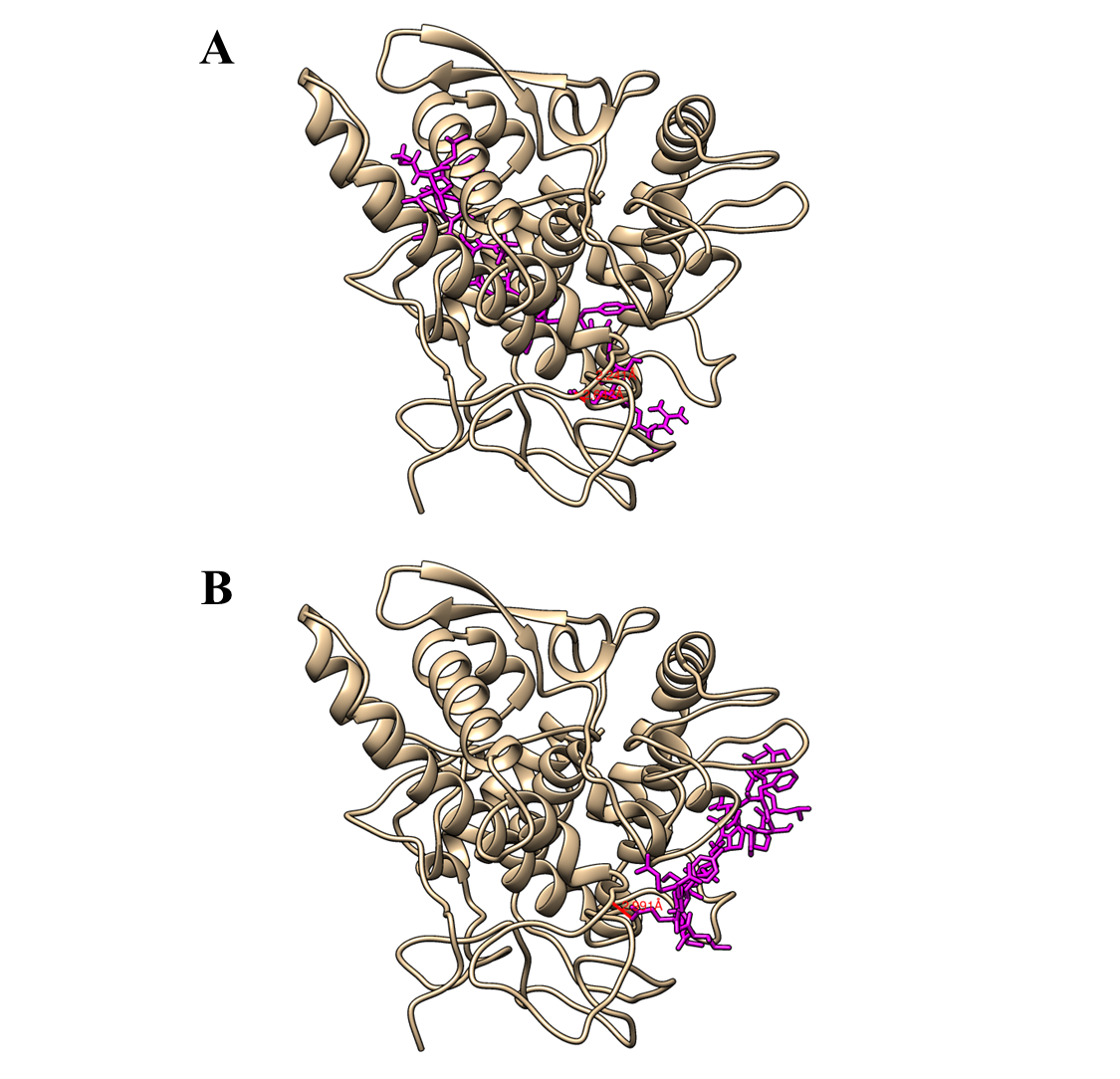


**Figure S4** Molecular docking of E2-12 peptide to the tyrosinase (PDB: 2Y9X) from GalaxyPepDock (A) and HPEPDOCK (B). The tyrosinase is shaded in gold, and the peptide sequences are colored in pink. The hydrogen bonds are shown as red lines.


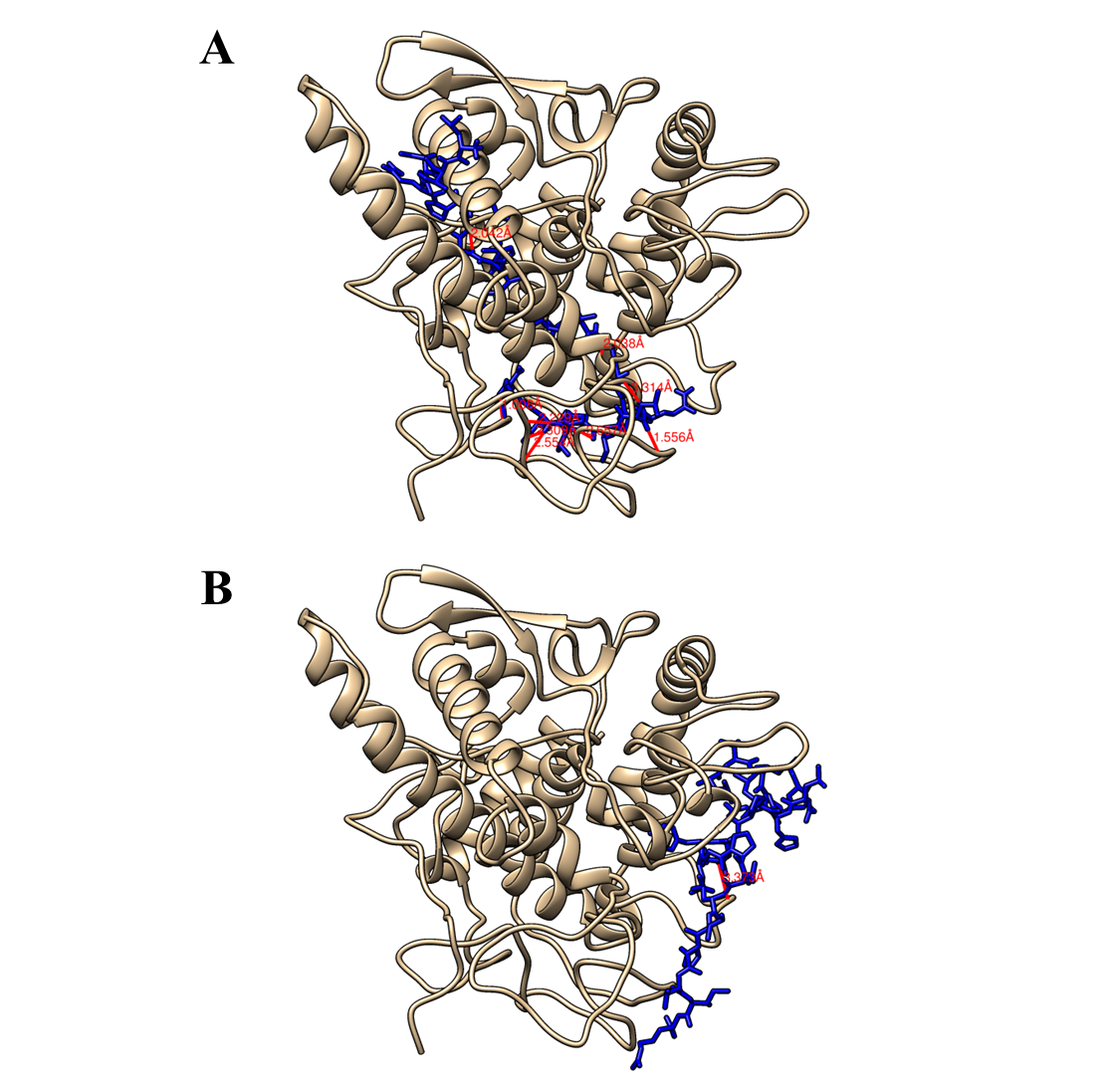


**Figure S5** Molecular docking of E3-6 peptide to the tyrosinase (PDB: 2Y9X) from GalaxyPepDock (A) and HPEPDOCK (B). The tyrosinase is shaded in gold, and the peptide sequences are colored in blue. The hydrogen bonds are shown as red lines.


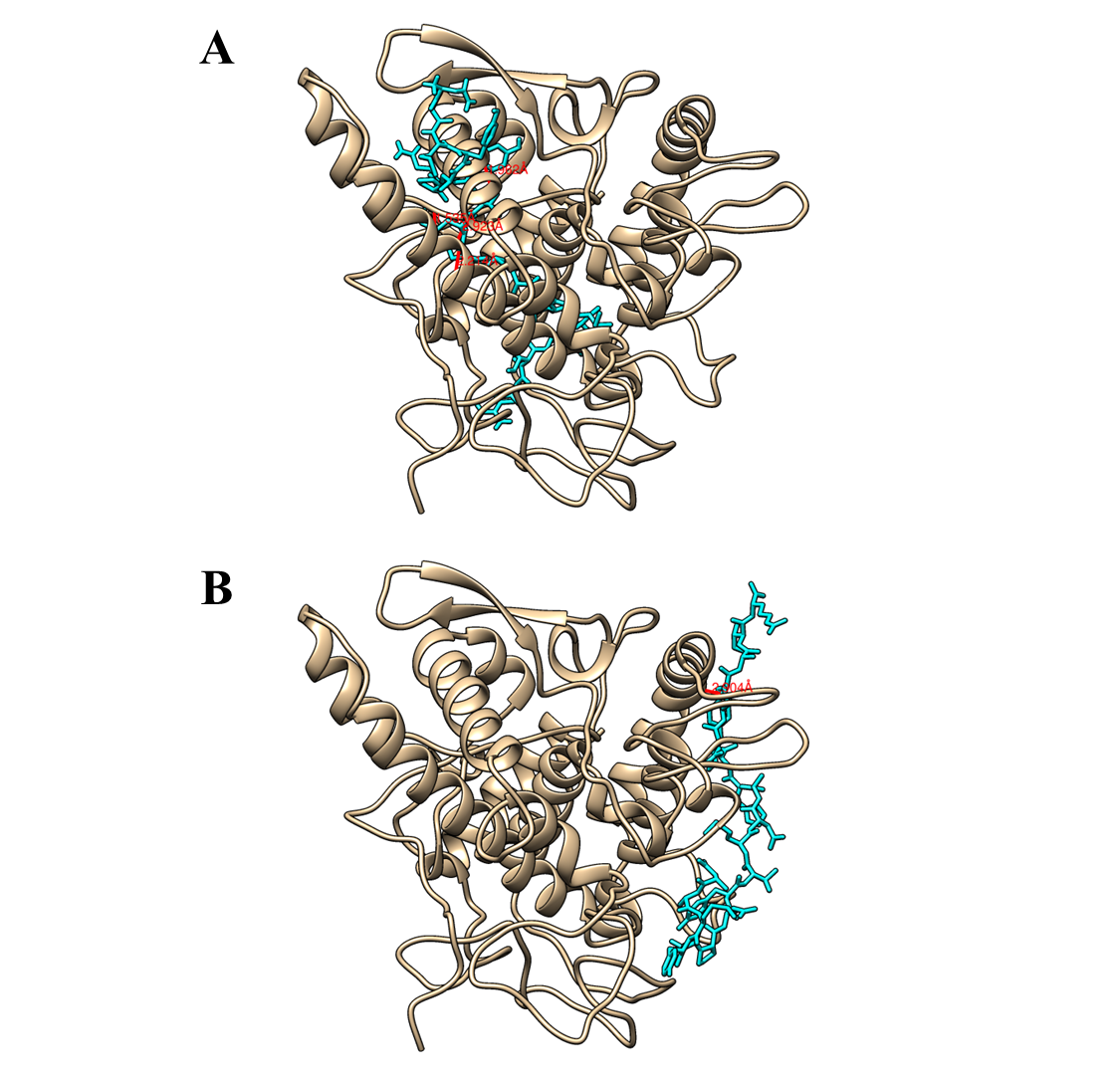


**Figure S6** Molecular docking of E3-38 peptide to the tyrosinase (PDB: 2Y9X) from GalaxyPepDock (A) and HPEPDOCK (B). The tyrosinase is shaded in gold, and the peptide sequences are colored in cyan. The hydrogen bonds are shown as red lines.

### **Supplementary Tables**

**Table S1** Cross-validation results of 130 single feature-based models developed using 13 different ML algorithms and 10 feature descriptors.

| **Feature** | **Method** | **ACC** | **Sn** | **Sp** | **MCC** | **AUC** | **Parameter** |
| --- | --- | --- | --- | --- | --- | --- | --- |
| AAC | ADA | 0.831 | 0.821 | 0.839 | 0.665 | 0.915 | 20 |
|  | DT | 0.745 | 0.724 | 0.764 | 0.492 | 0.744 | NA |
|  | ET | 0.830 | 0.810 | 0.849 | 0.665 | 0.921 | 200 |
|  | KNN | 0.698 | 0.764 | 0.631 | 0.403 | 0.697 | NA |
|  | LGBM | 0.830 | 0.799 | 0.859 | 0.666 | 0.884 | 50 |
|  | LR | 0.759 | 0.716 | 0.803 | 0.527 | 0.831 | 0.1 |
|  | MLP | 0.802 | 0.793 | 0.812 | 0.612 | 0.831 | 20 |
|  | NB | 0.754 | 0.745 | 0.765 | 0.517 | 0.832 | NA |
|  | PLS | 0.749 | 0.706 | 0.793 | 0.506 | 0.835 | NA |
|  | RF | 0.839 | 0.820 | 0.857 | 0.681 | 0.916 | 50 |
|  | SVMLN | 0.764 | 0.764 | 0.765 | 0.534 | 0.843 | 32 |
|  | SVMRBF | 0.778 | 0.801 | 0.754 | 0.563 | 0.869 | 1 |
|  | XGB | 0.826 | 0.819 | 0.831 | 0.656 | 0.895 | 200 |
| APAAC | ADA | 0.802 | 0.792 | 0.811 | 0.607 | 0.828 | 50 |
|  | DT | 0.713 | 0.708 | 0.715 | 0.427 | 0.712 | NA |
|  | ET | 0.849 | 0.851 | 0.849 | 0.705 | 0.899 | 100 |
|  | KNN | 0.760 | 0.691 | 0.829 | 0.530 | 0.760 | NA |
|  | LGBM | 0.811 | 0.809 | 0.812 | 0.630 | 0.859 | 20 |
|  | LR | 0.835 | 0.840 | 0.832 | 0.685 | 0.882 | 100 |
|  | MLP | 0.858 | 0.868 | 0.848 | 0.721 | 0.912 | 20 |
|  | NB | 0.778 | 0.784 | 0.774 | 0.568 | 0.821 | NA |
|  | PLS | 0.768 | 0.725 | 0.812 | 0.547 | 0.840 | NA |
|  | RF | 0.826 | 0.803 | 0.849 | 0.655 | 0.896 | 200 |
|  | SVMLN | 0.873 | 0.916 | 0.831 | 0.755 | 0.891 | 32 |
|  | SVMRBF | 0.859 | 0.870 | 0.850 | 0.731 | 0.919 | 4 |
|  | XGB | 0.807 | 0.810 | 0.801 | 0.621 | 0.865 | 100 |
| DDE | ADA | 0.859 | 0.841 | 0.879 | 0.728 | 0.899 | 200 |
|  | DT | 0.797 | 0.813 | 0.784 | 0.602 | 0.798 | NA |
|  | ET | 0.850 | 0.832 | 0.869 | 0.706 | 0.888 | 100 |
|  | KNN | 0.755 | 0.831 | 0.681 | 0.521 | 0.756 | NA |
|  | LGBM | 0.858 | 0.859 | 0.860 | 0.727 | 0.899 | 200 |
|  | LR | 0.713 | 0.748 | 0.679 | 0.437 | 0.741 | 10 |
|  | MLP | 0.708 | 0.737 | 0.679 | 0.423 | 0.738 | 200 |
|  | NB | 0.703 | 0.897 | 0.509 | 0.443 | 0.703 | NA |
|  | PLS | 0.600 | 0.457 | 0.745 | 0.212 | 0.621 | NA |
|  | RF | 0.859 | 0.813 | 0.907 | 0.726 | 0.891 | 20 |
|  | SVMLN | 0.708 | 0.746 | 0.671 | 0.426 | 0.721 | 2 |
|  | SVMRBF | 0.807 | 0.755 | 0.859 | 0.618 | 0.790 | 2 |
|  | XGB | 0.849 | 0.859 | 0.842 | 0.708 | 0.887 | 200 |
| DPC | ADA | 0.610 | 0.802 | 0.419 | 0.232 | 0.660 | 100 |
|  | DT | 0.609 | 0.672 | 0.551 | 0.258 | 0.611 | NA |
|  | ET | 0.689 | 0.830 | 0.549 | 0.396 | 0.764 | 50 |
|  | KNN | 0.642 | 0.776 | 0.512 | 0.313 | 0.644 | NA |
|  | LGBM | 0.481 | 0.600 | 0.400 | 0.000 | 0.500 | 20 |
|  | LR | 0.723 | 0.747 | 0.701 | 0.457 | 0.790 | 1 |
|  | MLP | 0.732 | 0.775 | 0.690 | 0.472 | 0.749 | 20 |
|  | NB | 0.671 | 0.597 | 0.745 | 0.351 | 0.671 | NA |
|  | PLS | 0.718 | 0.775 | 0.663 | 0.444 | 0.715 | NA |
|  | RF | 0.684 | 0.840 | 0.532 | 0.386 | 0.782 | 100 |
|  | SVMLN | 0.708 | 0.746 | 0.672 | 0.428 | 0.776 | 1 |
|  | SVMRBF | 0.713 | 0.688 | 0.737 | 0.444 | 0.772 | 2 |
|  | XGB | 0.618 | 0.295 | 0.941 | 0.319 | 0.646 | 50 |
| Estate | ADA | 0.788 | 0.735 | 0.843 | 0.593 | 0.859 | 20 |
|  | DT | 0.807 | 0.801 | 0.812 | 0.620 | 0.830 | NA |
|  | ET | 0.807 | 0.792 | 0.822 | 0.621 | 0.844 | 200 |
|  | KNN | 0.595 | 0.963 | 0.225 | 0.284 | 0.594 | NA |
|  | LGBM | 0.807 | 0.764 | 0.851 | 0.622 | 0.861 | 50 |
|  | LR | 0.807 | 0.773 | 0.843 | 0.623 | 0.860 | 0.1 |
|  | MLP | 0.807 | 0.792 | 0.823 | 0.622 | 0.852 | 100 |
|  | NB | 0.651 | 0.365 | 0.934 | 0.385 | 0.831 | NA |
|  | PLS | 0.798 | 0.735 | 0.861 | 0.607 | 0.852 | NA |
|  | RF | 0.788 | 0.755 | 0.822 | 0.584 | 0.846 | 200 |
|  | SVMLN | 0.793 | 0.783 | 0.804 | 0.593 | 0.852 | 4 |
|  | SVMRBF | 0.807 | 0.755 | 0.860 | 0.624 | 0.851 | 1 |
|  | XGB | 0.807 | 0.773 | 0.842 | 0.622 | 0.850 | 20 |
| FP4 | ADA | 0.750 | 0.733 | 0.766 | 0.518 | 0.847 | 200 |
|  | DT | 0.792 | 0.801 | 0.783 | 0.590 | 0.804 | NA |
|  | ET | 0.797 | 0.811 | 0.783 | 0.603 | 0.856 | 100 |
|  | KNN | 0.698 | 0.887 | 0.511 | 0.440 | 0.699 | NA |
|  | LGBM | 0.797 | 0.810 | 0.783 | 0.603 | 0.862 | 50 |
|  | LR | 0.793 | 0.735 | 0.852 | 0.598 | 0.852 | 0.01 |
|  | MLP | 0.792 | 0.791 | 0.792 | 0.597 | 0.856 | 50 |
|  | NB | 0.684 | 0.478 | 0.887 | 0.415 | 0.823 | NA |
|  | PLS | 0.783 | 0.743 | 0.823 | 0.573 | 0.859 | NA |
|  | RF | 0.802 | 0.754 | 0.848 | 0.612 | 0.870 | 100 |
|  | SVMLN | 0.812 | 0.782 | 0.842 | 0.631 | 0.859 | 1 |
|  | SVMRBF | 0.802 | 0.754 | 0.848 | 0.618 | 0.868 | 8 |
|  | XGB | 0.816 | 0.762 | 0.869 | 0.643 | 0.870 | 20 |
| MACCS | ADA | 0.817 | 0.811 | 0.822 | 0.645 | 0.870 | 20 |
|  | DT | 0.760 | 0.755 | 0.762 | 0.533 | 0.757 | NA |
|  | ET | 0.811 | 0.811 | 0.807 | 0.628 | 0.872 | 50 |
|  | KNN | 0.769 | 0.859 | 0.679 | 0.551 | 0.769 | NA |
|  | LGBM | 0.816 | 0.821 | 0.809 | 0.643 | 0.892 | 50 |
|  | LR | 0.817 | 0.820 | 0.813 | 0.648 | 0.880 | 10 |
|  | MLP | 0.792 | 0.810 | 0.771 | 0.594 | 0.889 | 50 |
|  | NB | 0.717 | 0.585 | 0.851 | 0.464 | 0.827 | NA |
|  | PLS | 0.760 | 0.735 | 0.785 | 0.529 | 0.860 | NA |
|  | RF | 0.797 | 0.782 | 0.809 | 0.599 | 0.897 | 200 |
|  | SVMLN | 0.798 | 0.822 | 0.775 | 0.612 | 0.879 | 2 |
|  | SVMRBF | 0.820 | 0.848 | 0.792 | 0.649 | 0.899 | 1 |
|  | XGB | 0.821 | 0.819 | 0.820 | 0.659 | 0.911 | 50 |
| PAAC | ADA | 0.817 | 0.802 | 0.831 | 0.646 | 0.848 | 20 |
|  | DT | 0.788 | 0.811 | 0.763 | 0.583 | 0.787 | NA |
|  | ET | 0.863 | 0.877 | 0.848 | 0.733 | 0.902 | 100 |
|  | KNN | 0.759 | 0.728 | 0.793 | 0.529 | 0.760 | NA |
|  | LGBM | 0.864 | 0.869 | 0.859 | 0.732 | 0.895 | 50 |
|  | LR | 0.831 | 0.831 | 0.832 | 0.673 | 0.893 | 100 |
|  | MLP | 0.882 | 0.871 | 0.897 | 0.773 | 0.914 | 200 |
|  | NB | 0.778 | 0.775 | 0.784 | 0.566 | 0.835 | NA |
|  | PLS | 0.764 | 0.687 | 0.839 | 0.543 | 0.855 | NA |
|  | RF | 0.859 | 0.878 | 0.839 | 0.724 | 0.893 | 200 |
|  | SVMLN | 0.831 | 0.850 | 0.813 | 0.677 | 0.883 | 4 |
|  | SVMRBF | 0.863 | 0.879 | 0.849 | 0.735 | 0.910 | 2 |
|  | XGB | 0.835 | 0.841 | 0.830 | 0.678 | 0.879 | 20 |
| PCP | ADA | 0.820 | 0.827 | 0.808 | 0.650 | 0.882 | 50 |
|  | DT | 0.793 | 0.802 | 0.781 | 0.593 | 0.789 | NA |
|  | ET | 0.845 | 0.879 | 0.809 | 0.699 | 0.901 | 20 |
|  | KNN | 0.731 | 0.785 | 0.677 | 0.468 | 0.731 | NA |
|  | LGBM | 0.835 | 0.821 | 0.850 | 0.688 | 0.915 | 50 |
|  | LR | 0.722 | 0.690 | 0.758 | 0.457 | 0.801 | 0.01 |
|  | MLP | 0.764 | 0.765 | 0.761 | 0.536 | 0.830 | 200 |
|  | NB | 0.703 | 0.699 | 0.707 | 0.411 | 0.766 | NA |
|  | PLS | 0.722 | 0.679 | 0.765 | 0.452 | 0.803 | NA |
|  | RF | 0.835 | 0.822 | 0.849 | 0.678 | 0.915 | 100 |
|  | SVMLN | 0.745 | 0.688 | 0.803 | 0.500 | 0.824 | 32 |
|  | SVMRBF | 0.760 | 0.775 | 0.741 | 0.523 | 0.836 | 4 |
|  | XGB | 0.826 | 0.801 | 0.848 | 0.665 | 0.904 | 50 |
| PubChem | ADA | 0.826 | 0.834 | 0.821 | 0.660 | 0.880 | 50 |
|  | DT | 0.774 | 0.783 | 0.765 | 0.551 | 0.774 | NA |
|  | ET | 0.835 | 0.820 | 0.845 | 0.678 | 0.927 | 100 |
|  | KNN | 0.840 | 0.868 | 0.810 | 0.690 | 0.839 | NA |
|  | LGBM | 0.850 | 0.862 | 0.838 | 0.704 | 0.916 | 200 |
|  | LR | 0.859 | 0.850 | 0.867 | 0.725 | 0.916 | 1 |
|  | MLP | 0.873 | 0.850 | 0.895 | 0.749 | 0.923 | 20 |
|  | NB | 0.679 | 0.539 | 0.824 | 0.380 | 0.710 | NA |
|  | PLS | 0.769 | 0.782 | 0.757 | 0.549 | 0.841 | NA |
|  | RF | 0.844 | 0.827 | 0.855 | 0.699 | 0.929 | 50 |
|  | SVMLN | 0.878 | 0.887 | 0.866 | 0.762 | 0.915 | 1 |
|  | SVMRBF | 0.882 | 0.869 | 0.894 | 0.767 | 0.926 | 16 |
|  | XGB | 0.854 | 0.861 | 0.848 | 0.714 | 0.929 | 200 |

**Table S2** Independent test results of 130 single feature-based models developed using 13 different ML algorithms and 10 feature descriptors.

| **Feature** | **Method** | **ACC** | **Sn** | **Sp** | **MCC** | **AUC** |
| --- | --- | --- | --- | --- | --- | --- |
| AAC | ADA | 0.865 | 0.852 | 0.867 | 0.578 | 0.948 |
|  | DT | 0.817 | 0.852 | 0.812 | 0.500 | 0.832 |
|  | ET | 0.889 | 0.889 | 0.890 | 0.641 | 0.970 |
|  | KNN | 0.654 | 0.926 | 0.613 | 0.364 | 0.770 |
|  | LGBM | 0.861 | 0.889 | 0.856 | 0.586 | 0.954 |
|  | LR | 0.846 | 0.815 | 0.851 | 0.527 | 0.920 |
|  | MLP | 0.846 | 0.852 | 0.845 | 0.545 | 0.916 |
|  | NB | 0.827 | 0.815 | 0.829 | 0.496 | 0.907 |
|  | PLS | 0.870 | 0.778 | 0.884 | 0.554 | 0.914 |
|  | RF | 0.904 | 0.926 | 0.901 | 0.686 | 0.964 |
|  | SVMLN | 0.861 | 0.852 | 0.862 | 0.569 | 0.902 |
|  | SVMRBF | 0.846 | 0.889 | 0.840 | 0.562 | 0.949 |
|  | XGB | 0.889 | 0.926 | 0.884 | 0.656 | 0.963 |
| APAAC | ADA | 0.832 | 0.926 | 0.818 | 0.557 | 0.903 |
|  | DT | 0.832 | 0.852 | 0.829 | 0.522 | 0.840 |
|  | ET | 0.909 | 0.889 | 0.912 | 0.683 | 0.970 |
|  | KNN | 0.837 | 0.815 | 0.840 | 0.511 | 0.827 |
|  | LGBM | 0.856 | 0.852 | 0.856 | 0.561 | 0.932 |
|  | LR | 0.885 | 0.926 | 0.878 | 0.646 | 0.919 |
|  | MLP | 0.894 | 0.926 | 0.890 | 0.666 | 0.929 |
|  | NB | 0.798 | 0.778 | 0.801 | 0.436 | 0.826 |
|  | PLS | 0.841 | 0.815 | 0.845 | 0.519 | 0.880 |
|  | RF | 0.913 | 0.852 | 0.923 | 0.681 | 0.957 |
|  | SVMLN | 0.880 | 0.926 | 0.873 | 0.637 | 0.920 |
|  | SVMRBF | 0.889 | 0.963 | 0.878 | 0.671 | 0.970 |
|  | XGB | 0.875 | 0.852 | 0.878 | 0.596 | 0.953 |
| DDE | ADA | 0.822 | 0.889 | 0.812 | 0.525 | 0.868 |
|  | DT | 0.784 | 0.852 | 0.773 | 0.455 | 0.813 |
|  | ET | 0.841 | 0.852 | 0.840 | 0.537 | 0.905 |
|  | KNN | 0.620 | 0.926 | 0.575 | 0.336 | 0.750 |
|  | LGBM | 0.822 | 0.889 | 0.812 | 0.525 | 0.885 |
|  | LR | 0.712 | 0.630 | 0.724 | 0.254 | 0.768 |
|  | MLP | 0.630 | 0.741 | 0.613 | 0.240 | 0.690 |
|  | NB | 0.582 | 0.889 | 0.536 | 0.286 | 0.712 |
|  | PLS | 0.731 | 0.444 | 0.773 | 0.168 | 0.722 |
|  | RF | 0.875 | 0.926 | 0.867 | 0.628 | 0.895 |
|  | SVMLN | 0.654 | 0.778 | 0.635 | 0.281 | 0.746 |
|  | SVMRBF | 0.673 | 0.889 | 0.641 | 0.360 | 0.727 |
|  | XGB | 0.837 | 0.926 | 0.823 | 0.564 | 0.889 |
| DPC | ADA | 0.615 | 0.889 | 0.575 | 0.312 | 0.786 |
|  | DT | 0.534 | 0.852 | 0.486 | 0.229 | 0.669 |
|  | ET | 0.635 | 0.889 | 0.597 | 0.327 | 0.847 |
|  | KNN | 0.601 | 0.778 | 0.575 | 0.237 | 0.676 |
|  | LGBM | 0.130 | 1.000 | 0.000 | 0.000 | 0.500 |
|  | LR | 0.687 | 0.815 | 0.669 | 0.332 | 0.783 |
|  | MLP | 0.687 | 0.815 | 0.669 | 0.332 | 0.768 |
|  | NB | 0.702 | 0.593 | 0.718 | 0.224 | 0.649 |
|  | PLS | 0.683 | 0.815 | 0.663 | 0.328 | 0.759 |
|  | RF | 0.587 | 0.926 | 0.536 | 0.311 | 0.874 |
|  | SVMLN | 0.697 | 0.815 | 0.680 | 0.342 | 0.762 |
|  | SVMRBF | 0.827 | 0.556 | 0.867 | 0.364 | 0.798 |
|  | XGB | 0.865 | 0.296 | 0.950 | 0.302 | 0.686 |
| Estate | ADA | 0.889 | 0.815 | 0.901 | 0.610 | 0.915 |
|  | DT | 0.788 | 0.926 | 0.768 | 0.499 | 0.884 |
|  | ET | 0.812 | 0.926 | 0.796 | 0.530 | 0.905 |
|  | KNN | 0.303 | 1.000 | 0.199 | 0.177 | 0.599 |
|  | LGBM | 0.880 | 0.815 | 0.890 | 0.590 | 0.935 |
|  | LR | 0.861 | 0.815 | 0.867 | 0.552 | 0.905 |
|  | MLP | 0.856 | 0.815 | 0.862 | 0.544 | 0.921 |
|  | NB | 0.904 | 0.444 | 0.972 | 0.511 | 0.861 |
|  | PLS | 0.870 | 0.815 | 0.878 | 0.571 | 0.889 |
|  | RF | 0.837 | 0.889 | 0.829 | 0.547 | 0.929 |
|  | SVMLN | 0.851 | 0.815 | 0.856 | 0.535 | 0.914 |
|  | SVMRBF | 0.856 | 0.815 | 0.862 | 0.544 | 0.904 |
|  | XGB | 0.865 | 0.815 | 0.873 | 0.561 | 0.923 |
| FP4 | ADA | 0.827 | 0.815 | 0.829 | 0.496 | 0.909 |
|  | DT | 0.817 | 0.926 | 0.801 | 0.537 | 0.853 |
|  | ET | 0.817 | 0.963 | 0.796 | 0.555 | 0.896 |
|  | KNN | 0.572 | 0.963 | 0.514 | 0.322 | 0.738 |
|  | LGBM | 0.894 | 0.852 | 0.901 | 0.636 | 0.957 |
|  | LR | 0.837 | 0.815 | 0.840 | 0.511 | 0.905 |
|  | MLP | 0.913 | 0.852 | 0.923 | 0.681 | 0.950 |
|  | NB | 0.851 | 0.481 | 0.906 | 0.371 | 0.833 |
|  | PLS | 0.846 | 0.778 | 0.856 | 0.510 | 0.912 |
|  | RF | 0.827 | 0.963 | 0.807 | 0.568 | 0.928 |
|  | SVMLN | 0.837 | 0.889 | 0.829 | 0.547 | 0.907 |
|  | SVMRBF | 0.904 | 0.852 | 0.912 | 0.657 | 0.933 |
|  | XGB | 0.889 | 0.852 | 0.895 | 0.625 | 0.934 |
| MACCS | ADA | 0.856 | 0.926 | 0.845 | 0.595 | 0.913 |
|  | DT | 0.764 | 0.815 | 0.757 | 0.413 | 0.788 |
|  | ET | 0.841 | 0.889 | 0.834 | 0.554 | 0.949 |
|  | KNN | 0.745 | 0.889 | 0.724 | 0.430 | 0.806 |
|  | LGBM | 0.851 | 0.889 | 0.845 | 0.570 | 0.934 |
|  | LR | 0.861 | 0.889 | 0.856 | 0.586 | 0.928 |
|  | MLP | 0.880 | 0.852 | 0.884 | 0.606 | 0.946 |
|  | NB | 0.837 | 0.556 | 0.878 | 0.381 | 0.858 |
|  | PLS | 0.841 | 0.852 | 0.840 | 0.537 | 0.904 |
|  | RF | 0.837 | 0.889 | 0.829 | 0.547 | 0.957 |
|  | SVMLN | 0.856 | 0.926 | 0.845 | 0.595 | 0.936 |
|  | SVMRBF | 0.865 | 0.889 | 0.862 | 0.595 | 0.938 |
|  | XGB | 0.817 | 0.889 | 0.807 | 0.519 | 0.937 |
| PAAC | ADA | 0.856 | 0.889 | 0.851 | 0.578 | 0.913 |
|  | DT | 0.875 | 0.815 | 0.884 | 0.580 | 0.849 |
|  | ET | 0.923 | 0.926 | 0.923 | 0.731 | 0.978 |
|  | KNN | 0.894 | 0.889 | 0.895 | 0.651 | 0.892 |
|  | LGBM | 0.870 | 0.889 | 0.867 | 0.603 | 0.962 |
|  | LR | 0.880 | 0.926 | 0.873 | 0.637 | 0.931 |
|  | MLP | 0.870 | 0.963 | 0.856 | 0.636 | 0.960 |
|  | NB | 0.784 | 0.704 | 0.796 | 0.378 | 0.814 |
|  | PLS | 0.846 | 0.778 | 0.856 | 0.510 | 0.865 |
|  | RF | 0.870 | 0.926 | 0.862 | 0.620 | 0.968 |
|  | SVMLN | 0.870 | 0.926 | 0.862 | 0.620 | 0.915 |
|  | SVMRBF | 0.899 | 1.000 | 0.884 | 0.705 | 0.970 |
|  | XGB | 0.870 | 0.889 | 0.867 | 0.603 | 0.943 |
| PCP | ADA | 0.837 | 0.741 | 0.851 | 0.475 | 0.850 |
|  | DT | 0.750 | 0.815 | 0.740 | 0.396 | 0.795 |
|  | ET | 0.832 | 0.852 | 0.829 | 0.522 | 0.935 |
|  | KNN | 0.697 | 0.741 | 0.691 | 0.301 | 0.716 |
|  | LGBM | 0.880 | 0.889 | 0.878 | 0.621 | 0.943 |
|  | LR | 0.784 | 0.778 | 0.785 | 0.417 | 0.803 |
|  | MLP | 0.784 | 0.852 | 0.773 | 0.455 | 0.878 |
|  | NB | 0.716 | 0.704 | 0.718 | 0.300 | 0.776 |
|  | PLS | 0.760 | 0.778 | 0.757 | 0.388 | 0.840 |
|  | RF | 0.822 | 0.741 | 0.834 | 0.452 | 0.921 |
|  | SVMLN | 0.793 | 0.778 | 0.796 | 0.430 | 0.839 |
|  | SVMRBF | 0.731 | 0.778 | 0.724 | 0.355 | 0.872 |
|  | XGB | 0.841 | 0.778 | 0.851 | 0.501 | 0.924 |
| PubChem | ADA | 0.880 | 0.963 | 0.867 | 0.653 | 0.965 |
|  | DT | 0.889 | 0.889 | 0.890 | 0.641 | 0.889 |
|  | ET | 0.885 | 0.926 | 0.878 | 0.646 | 0.945 |
|  | KNN | 0.803 | 0.889 | 0.790 | 0.499 | 0.839 |
|  | LGBM | 0.865 | 0.963 | 0.851 | 0.628 | 0.975 |
|  | LR | 0.875 | 1.000 | 0.856 | 0.660 | 0.967 |
|  | MLP | 0.889 | 0.963 | 0.878 | 0.671 | 0.957 |
|  | NB | 0.812 | 0.667 | 0.834 | 0.400 | 0.769 |
|  | PLS | 0.803 | 0.926 | 0.785 | 0.517 | 0.870 |
|  | RF | 0.861 | 0.852 | 0.862 | 0.569 | 0.941 |
|  | SVMLN | 0.875 | 1.000 | 0.856 | 0.660 | 0.965 |
|  | SVMRBF | 0.880 | 1.000 | 0.862 | 0.669 | 0.965 |
|  | XGB | 0.889 | 0.963 | 0.878 | 0.671 | 0.978 |

**Table S3** Average cross-validation results of each feature encoding over 13 different ML methods.

| **Descriptor** | **ACC** | **Sn** | **Sp** | **MCC** | **AUC** |
| --- | --- | --- | --- | --- | --- |
| AAC | 0.785 | 0.776 | 0.794 | 0.576 | 0.847 |
| APAAC | 0.811 | 0.805 | 0.816 | 0.629 | 0.853 |
| DDE | 0.774 | 0.784 | 0.766 | 0.560 | 0.795 |
| DPC | 0.661 | 0.703 | 0.624 | 0.346 | 0.698 |
| Estate | 0.774 | 0.753 | 0.796 | 0.569 | 0.829 |
| FP4 | 0.778 | 0.757 | 0.799 | 0.572 | 0.840 |
| MACCS | 0.792 | 0.791 | 0.792 | 0.596 | 0.862 |
| PAAC | 0.826 | 0.823 | 0.829 | 0.661 | 0.866 |
| PCP | 0.777 | 0.772 | 0.781 | 0.563 | 0.838 |
| PubChem | 0.828 | 0.818 | 0.837 | 0.664 | 0.879 |
| AAC | 0.785 | 0.776 | 0.794 | 0.576 | 0.847 |
| APAAC | 0.811 | 0.805 | 0.816 | 0.629 | 0.853 |

**Table S4** Performance Evaluation of BLAST-based predictors using different *E*-values.

| **E-Value** | **ACC** | **Sn** | **Sp** | **MCC** |
| --- | --- | --- | --- | --- |
| 0.1 | 0.894 | 0.185 | 1.000 | 0.406 |
| 0.01 | 0.885 | 0.111 | 1.000 | 0.313 |
| 0.001 | 0.880 | 0.074 | 1.000 | 0.255 |
| 0.0001 | 0.875 | 0.037 | 1.000 | 0.180 |

**Table S5** Average cross-validation results of each feature encoding over 13 different ML methods.

| **Feature** | **TIP** | **non-TIP** | **Difference** | ***p*-value** |
| --- | --- | --- | --- | --- |
| SVMRBF-APAAC | 0.757 | 0.264 | 0.493 | 1.12E-35 |
| ET-APAAC | 0.742 | 0.269 | 0.473 | 4.08E-33 |
| PLS-APAAC | 0.628 | 0.332 | 0.296 | 6.53E-19 |
| ET-DDE | 0.787 | 0.188 | 0.599 | 6.86E-35 |
| XGB-MACCS | 0.779 | 0.241 | 0.538 | 4.64E-32 |
| LR-MACCS | 0.736 | 0.270 | 0.466 | 7.73E-29 |
| SVMLN-PubChem | 0.758 | 0.255 | 0.503 | 6.69E-40 |
| LGBM-PubChem | 0.821 | 0.186 | 0.635 | 3.74E-40 |
| MLP-PubChem | 0.826 | 0.150 | 0.677 | 9.80E-41 |
| PLS-Estate | 0.676 | 0.306 | 0.371 | 7.56E-23 |
| ADA-Estate | 0.508 | 0.494 | 0.013 | 8.85E-21 |
| LR-FP4 | 0.706 | 0.300 | 0.406 | 4.81E-25 |

**Table S6** Impact of top 20 informative features derived from SVMRBF-APAAC.

| **Feature** | **TIP** | **non-TIP** | **Difference** | ***p*-value** |
| --- | --- | --- | --- | --- |
| Pc1.A | 0.092 | 0.098 | -0.006 | 0.813073 |
| Pc1.R | 0.179 | 0.039 | 0.140 | 8.36E-07 |
| Pc1.N | 0.064 | 0.058 | 0.006 | 0.781168 |
| Pc1.D | 0.034 | 0.143 | -0.109 | 0.000314 |
| Pc1.C | 0.213 | 0.017 | 0.196 | 3.71E-12 |
| Pc1.Q | 0.049 | 0.074 | -0.025 | 0.238499 |
| Pc1.E | 0.032 | 0.069 | -0.037 | 0.048194 |
| Pc1.G | 0.040 | 0.047 | -0.007 | 0.689718 |
| Pc1.H | 0.039 | 0.087 | -0.048 | 0.049023 |
| Pc1.I | 0.110 | 0.097 | 0.012 | 0.701157 |
| Pc1.L | 0.088 | 0.137 | -0.049 | 0.037697 |
| Pc1.K | 0.076 | 0.091 | -0.015 | 0.589444 |
| Pc1.M | 0.052 | 0.025 | 0.027 | 0.165746 |
| Pc1.F | 0.082 | 0.043 | 0.040 | 0.023963 |
| Pc1.P | 0.081 | 0.110 | -0.029 | 0.247657 |
| Pc1.S | 0.137 | 0.080 | 0.057 | 0.057508 |
| Pc1.T | 0.070 | 0.037 | 0.033 | 0.101486 |
| Pc1.W | 0.068 | 0.034 | 0.034 | 0.151427 |
| Pc1.Y | 0.111 | 0.032 | 0.079 | 0.000284 |
| Pc1.V | 0.153 | 0.086 | 0.066 | 0.022383 |
| Pc2.Hydrophobicity.1 | 0.742 | 0.712 | 0.029 | 0.078126 |
| Pc2.Hydrophilicity.1 | 0.590 | 0.572 | 0.017 | 0.365425 |

**Table S7** List of peptides used for virtual screening along with their probabilistic scores (PS) obtained from TIPred.

| **Seq No.** | **Name^a^** | **Sequence** | **PS** |
| --- | --- | --- | --- |
| seq1 | A-2 | ISSSTLALFAALMLVAHAVAFR | 1.0000 |
| seq13 | E1-9 | YTIQQNGLHLPSYTNTPQLVYIVK | 1.0000 |
| seq42 | E2-12 | GLLLPSFLNAPMMFYVIQGR | 1.0000 |
| seq72 | E3-38 | NAMYAPQYTMNAHNIIYAIR | 1.0000 |
| seq70 | E3-6 | LTIQPNGLHLPSYTNGPQLIHVIR | 1.0000 |
| seq45 | E2-13 | GIHGAVIPGCPETFER | 0.9910 |
| seq43 | E2-42 | NGMMAPHFNLDSHSVIYVTR | 0.9819 |
| seq68 | E3-8 | GVLGTLFPGCAETFEEAQVSVGGGR | 0.9524 |
| seq71 | E3-16 | FYIAGNPHQEFPQSMMTQQGR | 0.9516 |
| seq14 | E1-2 | ALLSLSFCFFLLLQGTSAISR | 0.9516 |
| seq11 | E1-16 | EGDIVAIPAGVAYWSYNNGDQQLVFVSLLDTSNVNNQLDDNPR | 0.9431 |
| seq94 | V-43 | SGCGVYLVNLTAGSMMAPHLNPK | 0.9293 |
| seq97 | V-42 | NNYGWSIALDEFSYSPLR | 0.9232 |
| seq16 | E1-11 | GILGVTFPGCPETFEESQR | 0.9169 |
| seq69 | E3-1 | MASTPLLLSLSLCFLVLLHGCSAR | 0.9155 |
| seq74 | E3-35 | LTTVNSYNLPILSFLR | 0.8989 |
| seq41 | E2-2 | SSTSLLCFTLFSLLLSHACFAQIEQMPQR | 0.8964 |
| seq102 | V-48 | AGPMEFFGFTTSAR | 0.8920 |
| seq39 | E2-19 | EGDMVAMPAGVADWVYNNGDSPLVLIAFVDVGNQANQLDQFSR | 0.8759 |
| seq92 | V-3 | LTLLVLMLVLSYGVLGIMGFDEDEDWTR | 0.8747 |
| seq2 | A-11 | FEEEDEIENYSQHLDQCCSQLR | 0.8655 |
| seq106 | V-47 | YFPFCQIASR | 0.8552 |
| seq67 | E3-5 | VECEGGMIESWNPNHEQFQCAGVALLR | 0.8520 |
| seq48 | E2-52 | AMPDDVLANAFQISR | 0.8501 |
| seq91 | V-26 | IGTFQSFFLGGGTNPASILSGFDSEILENAFNVTHAELK | 0.8419 |
| seq78 | E3-52 | EESSLFTTSHQGIR | 0.8325 |
| seq23 | E1-44 | ISTVNSYNLPILR | 0.8314 |
| seq40 | E2-10 | VECEAGVSEYWDIQNTEDDELHCAGVETAR | 0.8278 |
| seq50 | E2-39 | LNTLNNYNLPILR | 0.8195 |
| seq66 | E3-14 | EGDIIAIPAGMAYWCNNDGDQPLITVNLIHIINNQNQLDMSPR | 0.8155 |
| seq99 | V-24 | ISAGSAFYLVNTGEGQR | 0.8124 |
| seq101 | V-25 | LHIICSFDTSESLR | 0.8049 |
| seq79 | E3-31 | DYNGLEENICTMR | 0.7770 |
| seq26 | E1-59 | EETVLLTSSTSSR | 0.7669 |
| seq29 | E1-49 | VQVVNHMGQK | 0.7464 |
| seq98 | V-17 | TLFLPQYLDSELTIFIR | 0.7457 |
| seq73 | E3-21 | GFSVNLIQEAFNVDSETAR | 0.7447 |
| seq95 | V-45 | GTGMIQVVYPNGTSAMNTEVK | 0.7213 |
| seq22 | E1-41 | YEANGLEETFCSMR | 0.7209 |
| seq18 | E1-51 | QGQIVTVPQNHAVVK | 0.7145 |
| seq75 | E3-42 | QGQALTVPQNFAVVK | 0.7047 |
| seq46 | E2-27 | ILAESFNVDTELAHK | 0.6771 |
| seq76 | E3-48 | AMPEDVIANSYQISR | 0.6735 |
| seq44 | E2-26 | ESGEQTPNGNIFSGFDTR | 0.6670 |
| seq103 | V-51 | GPELAAAFGLSLER | 0.6666 |
| seq19 | E1-55 | ALPEAVLANAFQISR | 0.6551 |
| seq77 | E3-3 | TAMYGDQNECQLNR | 0.6529 |
| seq3 | A-3 | TTITTVETDDVENYSR | 0.6445 |
| seq20 | E1-12 | GQGQGQSQGSQPDR | 0.6374 |
| seq96 | V-27 | EILSSQQEGPIVYIPDSR | 0.6333 |
| seq28 | E1-43 | ADVFTPQAGR | 0.6285 |
| seq93 | V-33 | IVGFHQGEEEEDEEELEEDINQEQNQK | 0.6197 |
| seq4 | A-17 | NIPSMCGMQPR | 0.6172 |
| seq81 | E3-34 | ADVFSPQAGR | 0.6157 |
| seq52 | E2-35 | DNGLEETFCTLR | 0.6097 |
| seq17 | E1-18 | FYLAGNPEDEFEQLR | 0.5961 |
| seq49 | E2-56 | DEISVFSPSSQQTR | 0.5914 |
| seq25 | E1-53 | TNDNAWVSPLAGR | 0.5423 |
| seq47 | E2-46 | EGQIFVVPQNFAVVK | 0.5135 |
| seq24 | E1-52 | QASSDGFEWVSFK | 0.4793 |
| seq80 | E3-20 | EQEGLPNNVFR | 0.4530 |
| seq100 | V-53 | ESVILPTSAASPPVK | 0.4309 |
| seq53 | E2-48 | ASAQGFEWIAVK | 0.3447 |
| seq27 | E1-32 | GTLDLVSPLR | 0.3144 |
| seq5 | A-14 | CPALEMEIQK | 0.3052 |
| seq105 | V-40 | SPDSYNLYDGK | 0.3026 |
| seq51 | E2-16 | SEGASSDEQHQK | 0.2964 |
| seq12 | E1-60 | VEAEAGLIESWNPNHSQFQCAGVAVVR | 0.1942 |
| seq54 | E2-32 | GEDLQIIAPSR | 0.1942 |
| seq15 | E1-61 | NAIYTPHWNVSAHSVMYVLR | 0.1942 |
| seq21 | E1-62 | YPEEAFNVDSETVK | 0.1942 |
| seq55 | E2-57 | PSQADIFNPR | 0.1942 |
| seq104 | V-54 | PQFLVGASSILR | 0.1942 |

^a^The initial letter of hempseed peptides was named according to the origin protein; A (albumin), E1 (edestin1), E2 (edestin2), E3 (edestin3), and V (vicilin).

**Table S8** List of hydrogen bonds observed from the molecular docking of peptides to the crystal structure of the tyrosinase (PDB: 2Y9X).

| **GalaxyPepDock server** | | | | **HPEPDOCK server** | | | |
| --- | --- | --- | --- | --- | --- | --- | --- |
| **Name** | **Peptide Residues** | **Tyrosinase residues** | **Distance (Å)** | **Name** | **Peptide Residues** | **Tyrosinase residues** | **Distance (Å)** |
| A-2 | ALA 18 | ARG 20 | 2.908 | A-2 | ALA 20 | TYR 65 | 1.943 |
|  | LEU 8 | ARG 38 | 2.826 |  |  |  |  |
|  | SER 2 | THR 117 | 2.978 |  |  |  |  |
|  | SER 3 | ASP 164 | 3.300 |  |  |  |  |
|  | ALA 11 | VAL 37 | 2.942 |  |  |  |  |
|  | MET 13 | TYR 36 | 3.012 |  |  |  |  |
|  | VAL 19 | HIS 94 | 3.088 |  |  |  |  |
|  | ALA 20 | HIS 296 | 2.979 |  |  |  |  |
|  | ARG 22 | THR 92 | 2.865 |  |  |  |  |
|  | ARG 22 | SER 352 | 2.915 |  |  |  |  |
| E1-9 | PRO 17 | ARG 20 | 3.065 | E1-9 | TYR 13 | HIS 85 | 3.316 |
|  | LYS 24 | GLY 86 | 2.919 |  | GLN 18 | THR 187 | 3.339 |
|  | ASN 15 | GLN 133 | 2.771 |  | TYR 13 | HIS 85 | 3.316 |
|  | GLN 5 | ASN 166 | 2.926 |  |  |  |  |
|  | THR 2 | THR 168 | 3.052 |  |  |  |  |
|  | GLN 4 | GLU 171 | 3.264 |  |  |  |  |
|  | LEU 19 | HIS 296 | 3.053 |  |  |  |  |
|  | THR 16 | CYS 297 | 2.891 |  |  |  |  |
|  | PRO 17 | CYS 297 | 3.204 |  |  |  |  |
|  | GLN 4 | LYS 169 | 3.117 |  |  |  |  |
|  | THR 14 | GLN 133 | 3.065 |  |  |  |  |
|  | THR 16 | GLU 98 | 2.880 |  |  |  |  |
|  | GLN 18 | GLU 102 | 2.886 |  |  |  |  |
|  | LEU 19 | HIS 94 | 3.204 |  |  |  |  |
|  | TYR 21 | HIS 263 | 3.047 |  |  |  |  |
|  | LYS 24 | SER 242 | 2.755 |  |  |  |  |
| E2-12 | ASN 9 | GLN 133 | 2.774 | E2-12 | GLN 18 | TYR 65 | 2.991 |
|  | PHE 7 | GLN 133 | 2.846 |  | TYR 15 | TYR 65 | 1.582 |
|  | ARG 20 | MET 319 | 2.929 |  |  |  |  |
|  | ARG 20 | ARG 321 | 2.842 |  |  |  |  |
|  | ARG 20 | ARG 321 | 3.549 |  |  |  |  |
|  | ARG 20 | ARG 321 | 2.858 |  |  |  |  |
|  | SER 6 | ALA 128 | 2.880 |  |  |  |  |
|  | MET 13 | GLU 98 | 3.121 |  |  |  |  |
| E3-6 | GLN 18 | HIS 85 | 2.815 | E3-6 | SER 12 | VAL 248 | 3.373 |
|  | LEU 8 | GLN 133 | 2.781 |  | ARG 24 | TYR 78 | 2.687 |
|  | ASN 6 | GLN 133 | 2.879 |  | PRO 11 | ARG 268 | 3.188 |
|  | ASN 15 | HIS 259 | 2.830 |  | ASN 15 | SER 282 | 2.587 |
|  | LEU 10 | CYS 297 | 3.559 |  |  |  |  |
|  | GLN 18 | ARG 321 | 2.902 |  |  |  |  |
|  | ILE 20 | LEU 327 | 3.048 |  |  |  |  |
|  | HIS 21 | GLU 340 | 2.884 |  |  |  |  |
|  | LEU 1 | GLN 41 | 3.041 |  |  |  |  |
|  | THR 2 | TYR 165 | 3.347 |  |  |  |  |
|  | GLN 4 | TRP 124 | 2.594 |  |  |  |  |
|  | SER 12 | GLU 98 | 2.931 |  |  |  |  |
|  | THR 14 | HIS 94 | 2.847 |  |  |  |  |
|  | ASN 15 | PHE 241 | 2.825 |  |  |  |  |
|  | GLY 16 | GLU 256 | 2.975 |  |  |  |  |
|  | GLN 18 | THR 84 | 2.945 |  |  |  |  |
|  | GLN 18 | HIS 85 | 3.113 |  |  |  |  |
|  | LEU 19 | GLU 322 | 3.400 |  |  |  |  |
|  | ARG 24 | SER 352 | 2.832 |  |  |  |  |
|  | ARG 24 | SER 352 | 2.965 |  |  |  |  |
| E3-38 | HIS 13 | ARG 95 | 2.952 | E3-38 | TYR 17 | ALA 219 | 2.904 |
|  | ASN 11 | GLN 133 | 2.942 |  | ALA 2 | TYR 65 | 2.869 |
|  | ASN 14 | HIS 296 | 2.691 |  | TYR 4 | TYR 78 | 2.968 |
|  | ALA 12 | CYS 297 | 2.938 |  | MET 10 | ARG 268 | 3.436 |
|  | GLN 7 | TRP 124 | 2.958 |  | MET 10 | ARG 268 | 2.962 |
|  | THR 9 | TYR 36 | 2.777 |  |  |  |  |
|  | ASN 11 | LEU 131 | 2.843 |  |  |  |  |
|  | ARG 21 | ASP 353 | 2.833 |  |  |  |  |
|  | ARG 21 | GLU 356 | 2.843 |  |  |  |  |
|  | ARG 21 | ASP 351 | 2.813 |  |  |  |  |

### **References**

[1] P. Charoenkwan, W. Chiangjong, C. Nantasenamat, M. M. Hasan, B. Manavalan, and W. Shoombuatong, "StackIL6: a stacking ensemble model for improving the prediction of IL-6 inducing peptides," *Briefings in Bioinformatics,* vol. 22, no. 6, p. bbab172, 2021.

[2] P. Charoenkwan, C. Nantasenamat, M. M. Hasan, M. A. Moni, B. Manavalan, and W. Shoombuatong, "StackDPPIV: A novel computational approach for accurate prediction of dipeptidyl peptidase IV (DPP-IV) inhibitory peptides," *Methods,* vol. 204, pp. 189-198, 2022.

[3] P. Charoenkwan, N. Schaduangrat, C. Nantasenamat, T. Piacham, and W. Shoombuatong, "iQSP: A Sequence-Based Tool for the Prediction and Analysis of Quorum Sensing Peptides Using Informative Physicochemical Properties," *International Journal of Molecular Sciences,* vol. 21, no. 1, p. 75, 2019.

[4] M. Azadpour, C. M. McKay, and R. L. Smith, "Estimating confidence intervals for information transfer analysis of confusion matrices," *The Journal of the Acoustical Society of America,* vol. 135, no. 3, pp. EL140-EL146, 2014.

[5] P. Charoenkwan *et al.*, "AMYPred-FRL is a novel approach for accurate prediction of amyloid proteins by using feature representation learning," *Scientific reports,* vol. 12, no. 1, pp. 1-14, 2022.

[6] P. Charoenkwan, N. Schaduangrat, M. A. Moni, B. Manavalan, and W. Shoombuatong, "SAPPHIRE: A stacking-based ensemble learning framework for accurate prediction of thermophilic proteins," *Computers in Biology and Medicine,* p. 105704, 2022.

[7] F.-Y. Dao, H. Lv, D. Zhang, Z.-M. Zhang, L. Liu, and H. Lin, "DeepYY1: a deep learning approach to identify YY1-mediated chromatin loops," *Briefings in bioinformatics,* vol. 22, no. 4, p. bbaa356, 2021.

[8] H. Yang *et al.*, "A comparison and assessment of computational method for identifying recombination hotspots in Saccharomyces cerevisiae," *Briefings in bioinformatics,* vol. 21, no. 5, pp. 1568-1580, 2020.

[9] F.-Y. Dao *et al.*, "Identify origin of replication in Saccharomyces cerevisiae using two-step feature selection technique," *Bioinformatics,* vol. 35, no. 12, pp. 2075-2083, 2019.

[10] W. Chen, H. Lv, F. Nie, and H. Lin, "i6mA-Pred: identifying DNA N6-methyladenine sites in the rice genome," *Bioinformatics,* vol. 35, no. 16, pp. 2796-2800, 2019.

[11] H. Lv, F.-Y. Dao, Z.-X. Guan, H. Yang, Y.-W. Li, and H. Lin, "Deep-Kcr: accurate detection of lysine crotonylation sites using deep learning method," *Briefings in Bioinformatics,* vol. 22, no. 4, p. bbaa255, 2021.

[12] Z.-C. Xu, P.-M. Feng, H. Yang, W.-R. Qiu, W. Chen, and H. Lin, "iRNAD: a computational tool for identifying D modification sites in RNA sequence," *Bioinformatics,* vol. 35, no. 23, pp. 4922-4929, 2019.
